# Better in the Near Infrared: Sulfonamide Perfluorinated-Phenyl Photosensitizers for Improved Simultaneous Targeted Photodynamic Therapy and Real-Time Fluorescence Imaging

**DOI:** 10.1021/acsami.4c11171

**Published:** 2024-09-14

**Authors:** Marta Warszyńska, Barbara Pucelik, Carolina S. Vinagreiro, Paweł Repetowski, Agata Barzowska, Dominik Barczyk, Fábio A. Schaberle, Amilcar Duque-Prata, Luis G. Arnaut, Mariette M. Pereira, Janusz M. Dąbrowski

**Affiliations:** † Faculty of Chemistry, Jagiellonian University, 30-387 Kraków, Poland; ‡ Doctoral School of Exact and Natural Sciences, Jagiellonian University, 30-348 Kraków, Poland; § Łukasiewicz Research Network−Kraków Institute of Technology, ul. Zakopiańska 73, 30-418 Kraków, Poland; ∥ CQC-IMS, Department of Chemistry, University of Coimbra, 3004-535 Coimbra, Portugal

**Keywords:** anticancer agents, bacteriochlorins, fluorescence imaging, organoids, photodynamic therapy (PDT), porphyrins, reactive oxygen species (ROS), rodent models, tumors

## Abstract

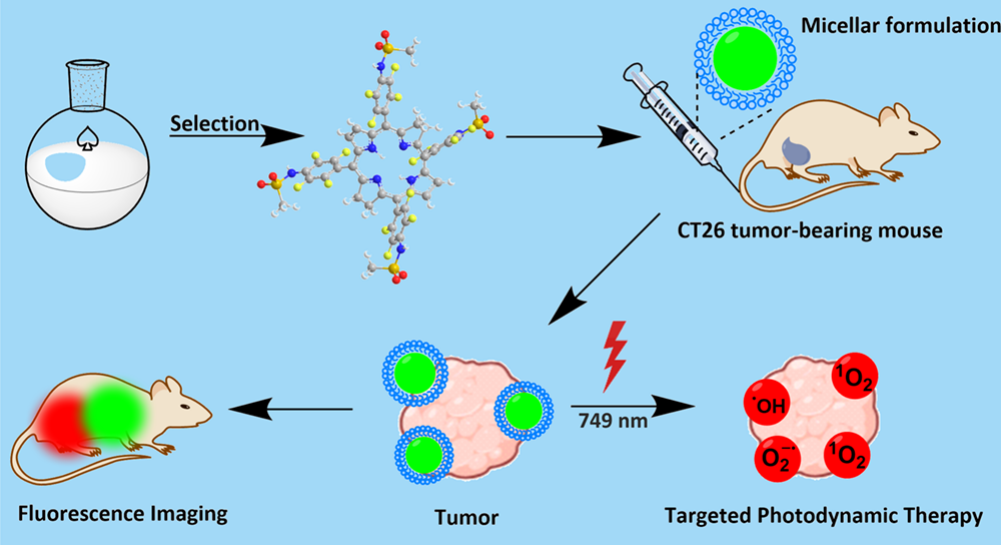

Tetraphenyloporphyrin derivatives are a useful scaffold for developing new pharmaceuticals for photodynamic therapy (PDT) and the photodiagnosis (PD) of cancer. We synthesized new sulfonamide fluorinated porphyrin derivatives and investigated their potential as photosensitizers and real-time bioimaging agents for cancer. We found that 5,10,15,20-tetrakis-[2′,3′,5′,6′-tetrafluoro-4′-methanesulfamidyl)phenyl]bacteriochlorin (**F**_**4**_**BMet**) has intense absorption and fluorescence in the near-infrared, efficiently generates singlet oxygen and hydroxyl radicals, has low toxicity in the dark, and high phototoxicity. We increased its bioavailability with encapsulation in Pluronic-based micelles, which also improved the photodynamic effect. **F**_**4**_**BMet** exhibits pH-dependent properties (lower pH promoted its aggregation), and a GlyGly buffer was used to effectively solubilize the compound. In vitro findings with 2D cell culture were complemented with human-induced pluripotent stem cell (hiPSC)-derived organoids. **F**_**4**_**BMet** in **P123** micelles showed enhanced efficacy compared to **F**_**4**_**BMet** in the **GlyGly** formulation. **F**_**4**_**BMet** was further evaluated in real-time bioimaging and PDT of BALB/c mice bearing CT26 tumors. After *i.v.* injection, the photosensitizer was visible in the tumor area 3 h after injection. The most successful therapeutic approach proved to be tumor-targeted PDT using P123-encapsulated **F**_**4**_**BMet** illuminated 24 h after administration with a light dose of 42 J/cm^2^, which led to a 30% long-term cure rate.

## Introduction

Combining the targeted therapy of cancer, characterized by an enhanced therapeutic index, with diagnostic properties for bioimaging into a single multimodal compound remains a challenge, but interest in such theranostic agents has grown in recent years. A variety of imaging techniques can be used, together with therapeutic protocols. One of these is combining photodynamic therapy (PDT) with noninvasive fluorescence imaging.^1,2^ Photosensitizers employed in PDT are especially suitable for multimodal techniques, as the majority of them feature red or near-infrared (NIR) fluorescence.^3^ The main challenge is to find the right balance between the optical (NIR absorption and fluorescence) and photochemical (generation of long-lasting triplet states) properties of the photosensitizer along with its increased selectivity to tumor tissue to enable its use both for noninvasive imaging and effective therapy.

PDT is a clinically approved, minimally invasive therapeutic procedure, entering the mainstream of oncology, with proven results in the treatment of various cancers.^4^ The mechanism of PDT relies on a combination of a photosensitizer molecule, light with an appropriate wavelength absorbed by the photosensitizer, and molecular oxygen to trigger photochemical and biological mechanisms processes that lead to primary tumor destruction and possibly long-lasting protection against metastasis.^5−9^

Tetrapyrrolic macrocycles, particularly those with NIR absorption, namely chlorins, bacteriochlorins, and phthalocyanines, have been largely explored as scaffolds for the development of more efficient photosensitizers for PDT of cancer or/and photodynamic inactivation (PDI) of microorganisms.^10−13^ These compounds combine flexibility for synthetic modulation, low dark toxicity, strong light absorption in the phototherapeutic window (600–850 nm), and high quantum yields of reactive oxygen species (ROS). ROS are generated according to two main types of photochemical reactions: Type I (electron transfer), leading to hydroxyl radicals, and Type II (energy transfer), resulting in singlet oxygen.^14^ These species are responsible for local oxidative stress that may lead to cell death—mostly apoptosis or necrosis.^15,16^ Photosensitizers must combine high quantum yields for singlet oxygen (Φ_Δ_) and/or oxygen radical generation, with reasonable photostability–i.e., low photodecomposition quantum yield (Φ_PD_). The usefulness of porphyrin derivatives as PDT photosensitizers was demonstrated by the number of pharmaceutical formulations clinically approved for PDT in Europe and in the US, such as those of Foscan (temoporfin),^17^ Photofrin (porfirmer sodium),^18^ Visudyne (verteporfin),^19^ and Tookad Soluble (padeliporfin),^20^ and in clinical trials, such as redaporfin.^7,8^

NIR photons, especially in the 700–900 nm region, are attractive for biomedical applications due to low background autofluorescence and deep tissue penetration. Therefore, potential optical imaging agents should be characterized by a high molar absorption coefficient in NIR, large fluorescence quantum yield, appropriate Stokes shift to separate excitation from emission, low toxicity, and low photodecomposition quantum yield (Φ_PD_).^21^ Porphyrinoids are widely used because their specific accumulation in tumor tissue is beneficial in real-time imaging during surgery or cancer diagnostics.^22−25^ Numerous studies have focused on the development of bacteriochlorin-based fluorescent contrasts.^26^ However, due to low stability, their structure must be modified, for instance, by adding other NIR fluorophores through the formation of energy-transfer dyads with chlorins,^27,28^ by adding naphtalimide,^29^ the addition of thioglycoside^30^ or by building nanoparticles in the form of self-assembled bacteriochlorin nanoparticles.^2,31,32^ Bacteriochlorins can also be conjugated with PEG to form nanogels to enhance their stability.^33^ Moreover, bacteriochlorin analogs called bacteriopurpurins show potential as bimodal agents for PDT and NIR fluorophores. Tetrapyrrolic and tetracyclic macrocycles can be modified to maximize the fluorescence quantum yield (Φ_F_), which changes their purpose to be solely used in bioimaging. Substitution of phthalocyanine with butoxyl groups and metallization with Si(OH)_2_ resulted in a compound with high Φ_F_, and good photostability, which was selectively accumulated in 4T1 tumors in BALB/c mice.^34^

We have previously demonstrated that halogenated tetraphenyl porphyrin derivatives are a convenient platform for developing stable photosensitizers with high singlet oxygen quantum yields (Φ_Δ_).^35,36^ Sulfonation of halogenated porphyrin derivatives followed by nucleophilic attack by water or amines gave access to large quantities of sulfonamide derivatives with a wide range of polarities.^36−38^ These derivatives are characterized by the presence of Ph-SO_2_NHR or Ph-SO_2_NR_2_ substituents in the *meso*-positions of the porphyrin ring, where the phenyl group is halogenated in *ortho*-positions. A tetraphenyl bacteriochlorin derivative with fluorine atoms in *ortho*-positions and Ph-SO_2_NHCH_3_ substituents in one *meta*-position of the phenyl groups, named redaporfin, has proven to be particularly phototoxic and suitable for PDT.^7,36,39,40^ However, it is the most efficient in vascular-targeted photodynamic therapy (V-PDT) with short drug to light interval (DLI = 15 min).^41^ In cellular-targeted PDT (C-PDT), redaporfin demonstrated clear effectiveness against control groups but failed to achieve any long-term cures in the murine model of colon cancer.^7,36,39,40^ Therefore, it remains challenging to find fluorinated sulfonamide bacteriochlorins for C-PDT that may also work as real-time fluorescence imaging agents.

There is also continued interest in the development of photosensitizers that respond to the tumor milieu. In particular, pH-responsiveness has been used for cancer-specific targeting because the extracellular pH of tumor tissues is more acidic (pH 6.5) than that of blood (pH 7.4).^42−45^ Previously, we described the pH-dependent profile of the Pluronic-P123-based formulation of redaporfin and found that the drug release behavior of this formulation is related to the low pH stimulus. The disorganization of the micellar structure is important for drug release in the acidic tumor microenvironment (TME).^46^ Moreover, this polymer-based formulation is safer than the Cremophor EL formulation for redaporfin in clinical practice.^41−46^

**Scheme 1:**
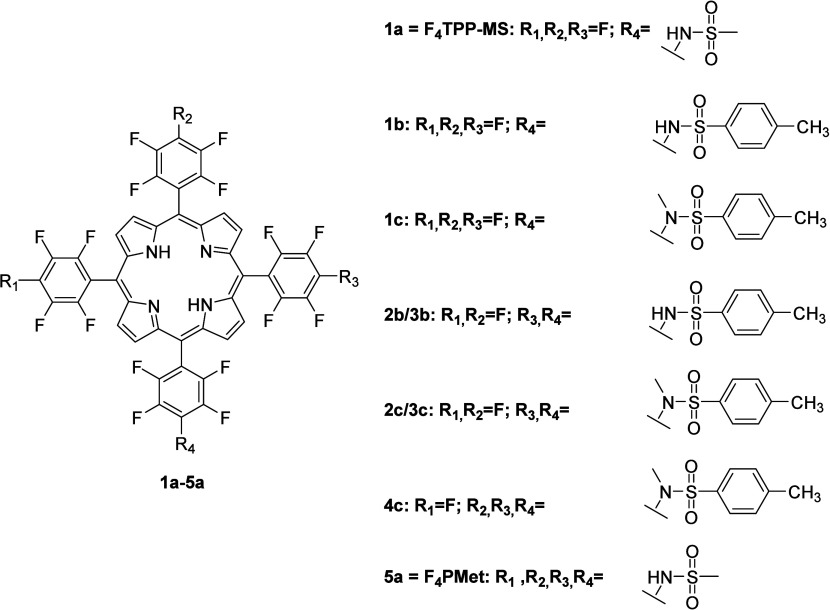
Schematic Presentation of the Chemical Structures of Various Porphyrins with Differently Situated Substituents, Previously Synthesized and Characterized,^47^ Being the Family of Photosensitizers from Which the Lead Compound Described in This Paper Is Derived

In this work, we explore molecules related to redaporfin, but featuring a different linker to the sulfonamide group (i.e., Ph-NHSO_2_R or Ph-NRSO_2_R) and a higher degree of fluorination of the phenyl groups.^47^ These changes in the chemical structure improve the solubility in biocompatible solvents and increase their oxidation potential, yielding more stable photosensitizers. In addition, as shown in Scheme 1, sulfonamide groups located in the *para*-positions of the phenyl ring avoid atropoisomers.^48^ Cytotoxic and phototoxic effects in vitro of the most promising photosensitizers were evaluated against A549 (human lung adenocarcinoma), B16–F10 (murine skin melanoma), and CT26 (murine colon carcinoma) cancer cells and on human-induced pluripotent stem cell (hiPSC)-derived colonic organoids. Those colonic organoids are an innovative model of colon cancer, as they provide a more fitting framework than traditional 2D cell cultures and have greater heterogeneity, which makes them a better representation of real tumors. The other benefit of organoids in preclinical studies is that research with such structures fits into the 3R (replacement, reduction, and refinement) principle. The pH-dependent behavior of the most promising bacteriochlorin, **F**_**4**_**BMet**, which was found to be highly soluble in buffered aqueous solution, motivated experiments with hiPSC-derived colonic organoids using micellar and glycylglycine (GlyGly) formulations. Subsequently, the potential of **F**_**4**_**BMet** for in vivo real-time fluorescence imaging in BALB/c mice bearing CT26 tumors was evaluated, and PDT efficacy was determined with several protocols (different formulations, drug-light intervals, and light doses) and compared with redaporfin (**F**_**2**_**BMet**).

## Results and Discussion

### Synthesis

The porphyrins presented in Scheme 1 were synthesized according to the previously described methodology.^47^ The first step of the synthesis was the preparation of 5,10,15,20-tetrakis(pentafluorophenyl)porphyrin by the condensation reaction of pyrrol with pentafluorobenzaldehyde in an acetic acid/nitrobenzene mixture with NaY as a Lewis acid catalyst. Sulfonamide porphyrins were prepared by reaction with appropriate sulfonamide and a base (for example, cesium carbonate). Mono- or tetrasubstituted products were obtained by changing the proportions among porphyrins, sulfonamide, and base. With this methodology, a library of several, already characterized porphyrins was obtained (Scheme 1).^47^

The F_4_PMet (Scheme 1, **5a**) showed biocompatibility with reasonable photostability, and adequate production of singlet oxygen led us to prepare its corresponding bacteriochlorin (**F**_**4**_**BMet**) to shift their absorption toward the phototherapeutic window.

**Scheme 2:**
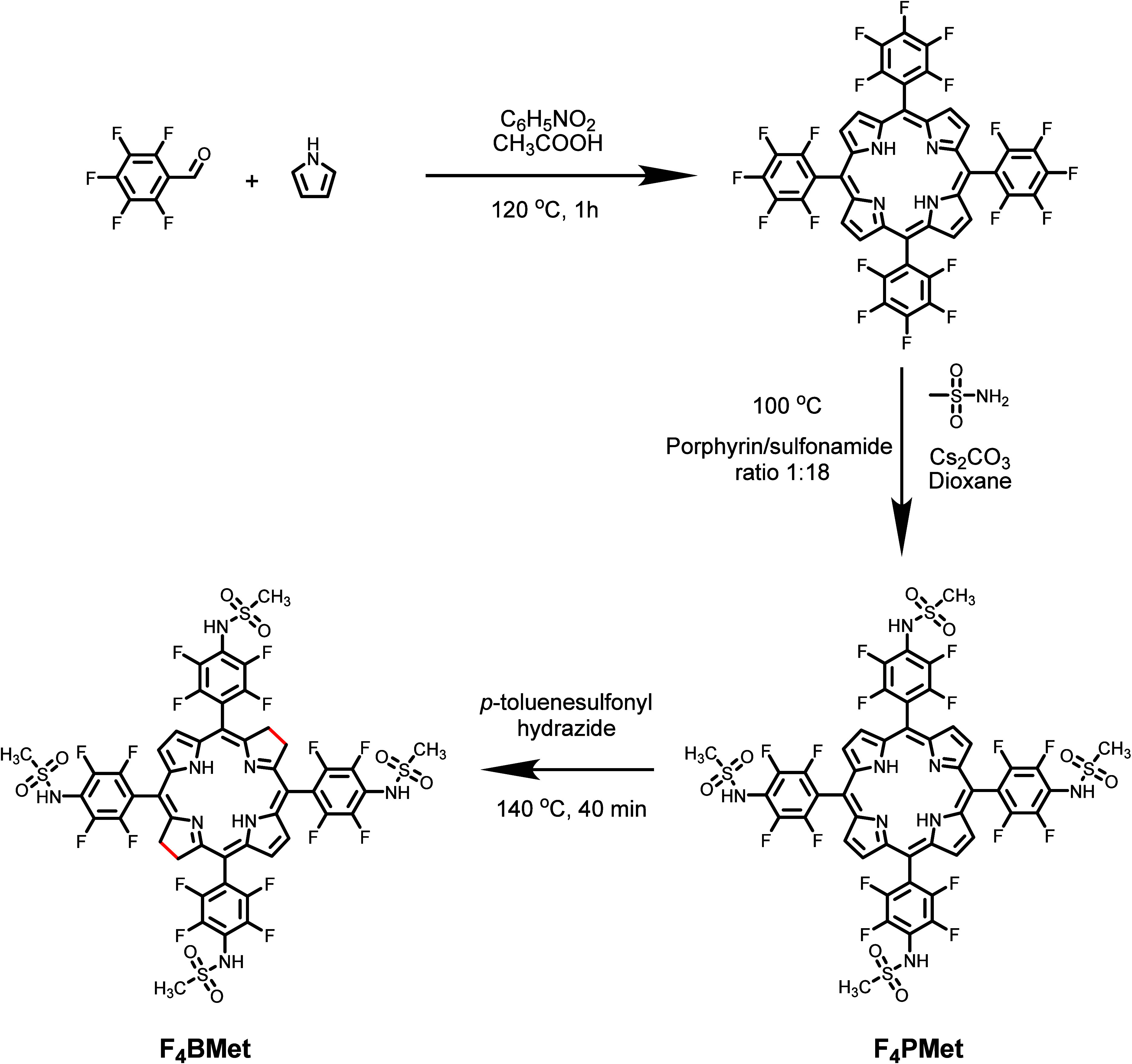
Schematic Illustration of the Synthesis of **F**_**4**_**BMet** Using *p*-Toluenesulfonylhydrazide According to the Solvent-Free Methodology

The 5,10,15,20-tetrakis-[2′,3′,5′,6′-tetrafluoro-4′-methanesulfamidyl)phenyl]bacteriochlorin **F**_**4**_**BMet** was synthesized through a hydrazide reduction using the solvent-free methodology.^36^ In a typical solid–solid reaction, the two solids, porphyrin **F**_**4**_**PMet** and an excess of *p*-toluenesulfonylhydrazide (1:40), are mixed together and heated up to 140 °C for 40 min in the absence of oxygen under vacuum (Scheme 2).^36^ After cooling to room temperature, the crude product is purified by silica gel column chromatography (dichloromethane/ethyl acetate 3:1), obtaining a new, corresponding bacteriochlorin **F**_**4**_**BMet** with a 70% yield.

### Optical and Photophysical Properties

The electronic absorption spectrum of the studied porphyrin is well understood, illustrating the typical features of these macrocycles, with the longest wavelength absorption bands situated between 635 and 644 nm and molar absorption coefficients (ε) ca. 1000 M^–1^ cm^–1^ (Figure S1, Supporting Information).^47^ The reduction of the two pyrroles on the opposite sides of the macrocycle (Scheme 2) increases the probability of the electronic transition at a longer wavelength, originating a Q_*y*_-band at 745 nm with ε = 1.31 × 10^5^ M^–1^ cm^–1^ in ethanol, consistent with the values of other bacteriochlorins reported.^49^

**Figure 1:**
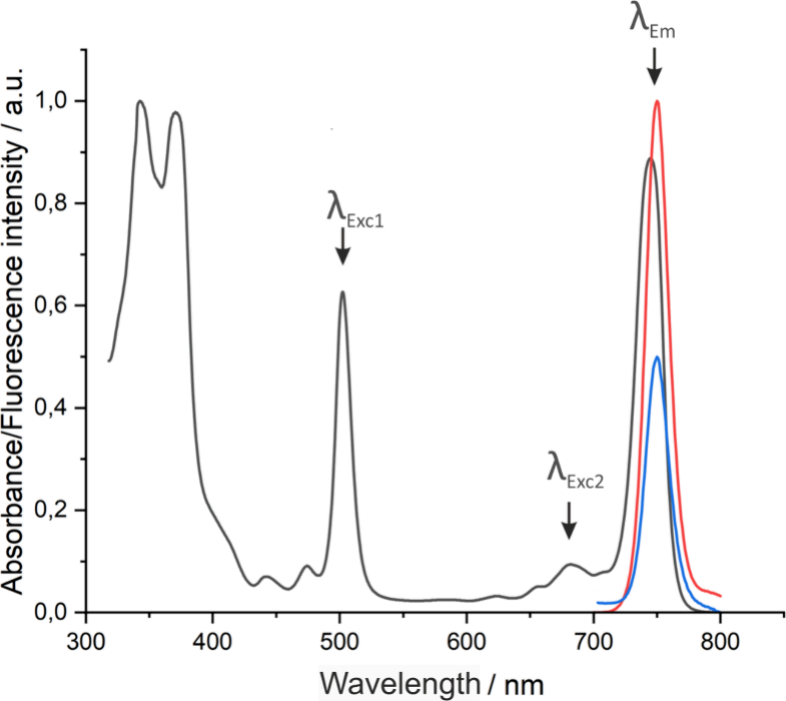
Normalized electronic absorption and emission spectra of the resulting bacteriochlorin **F**_**4**_**BMet** in ethanol. Fluorescence spectra were recorded after excitation with either λ_Exc1_ = 514 nm (red) or λ_Exc2_ = 680 nm (blue).

The electronic absorption spectrum of **F**_**4**_**BMet** shown in Figure 1 is a consequence of the change in the energy of the HOMO and LUMO orbitals. The analysis of the spatial distribution of HOMO and LUMO orbitals (Figures S2 and S3) indicates that the presence of fluorine atoms on the phenyl substituents does not affect their distribution. However, the fluorinated phenyl rings cause a slight reduction in the HOMO–LUMO energy gap, which is manifested in a bathochromic shift of the lowest energy absorption band. **F**_**4**_**BMet** exhibits an absorption band at 745 nm in ethanol, whereas redaporfin, which contains fluorine atoms in the *ortho* positions of the phenyl ring but hydrogen atoms in *para* and *meta* positions, absorbs at 743 nm in ethanol.^36^ This shift is even more pronounced in other solvents, as **F**_**4**_**BMet** features an absorption band at λ_max_ = 752 nm in DMSO and λ_max_ = 753 nm in the aqueous micellar formulation. The orbital energies are detailed in the Supporting Information (Tables S1–S3).

Additionally, Log *P*_OW_ was determined for **F**_**4**_**BMet** and the corresponding porphyrin (F_4_PMet) (Table S4, Figure S4, Supporting Information).

The fluorescence spectra of the porphyrins possess bands at ca. 644 nm, with a Stokes shift between 1 and 8 nm. The fluorescence quantum yields of the perfluorinated porphyrins **1a**–**5a** are in the range of 0.019–0.047 (Table 1). As expected from the internal heavy-atom effect, these values are lower than those of sulfonamide porphyrins with one (Φ_F_ = 0.096) or two (Φ_F_ = 0.049) fluorine atoms in the *ortho* positions of the phenyl rings previously described in the literature.^36^

**Table 1:** Optical and Photophysical Properties: Molar Absorption Coefficient (ε), Fluorescence Quantum Yield (Φ_F_), Triplet State Lifetimes (τ_T_), Singlet Oxygen Quantum Yield (Φ_Δ_), Photodecomposition Quantum Yield (Φ_PD_) of Perfluorinated Porphyrins - **1a** (**F**_**4**_**TPP-MS)**, **1b**, **1c**, **2b**/**3b**, **4c**, and **5a** (**F**_**4**_**PMet)** in Toluene,^47^ and Bacteriochlorin - **F**_**4**_**BMet** in Ethanol

photosensitizer	ε/M^–1^ cm^–1^	τ_T_ (O_2_)/μs	τ_T_ (N_2_)/μs	Φ_Δ_	Φ_F_	Φ_PD_/10^–6^	ref.
**F**_**4**_**TPP-MS/1a**	6.6 × 10^2^ (636 nm)	0.59 ± 0.01	45.14 ± 1.76	0.74 ± 0.08	0.0035	10	(47)
**1b**	8.2 × 10^2^ (637 nm)	0.59 ± 0.03	34.45 ± 3	0.70 ± 0.04	0.034	19	(47)
**1c**	8.3 × 10^2^ (636 nm)	0.67 ± 0.02	33.34 ± 0.38	0.66 ± 0.04	0.042	459	(47)
**2b/3b**	1.3 × 10^3^ (644 nm)	0.62 ± 0.02	40.99 ± 1.17	0.71 ± 0.05	0.047	23	(47)
**4c**	9.6 × 10^2^ (640 nm)	0.71 ± 0.008	45.82 ± 0.78	0.59 ± 0.05	0.030	2195	(47)
**F**_**4**_**PMet/5a**	6.3 × 10^2^ (638 nm)			0.70	0.019	48	(47)
**F**_**4**_**BMet**	1.31 × 10^5^ (745 nm)	0.27 ± 0.03	28.34 ± 4.68	0.36	0.20	18	this work

**F**_**4**_**BMet** is characterized by a larger Stokes shift than other bacteriochlorins and its fluorescence quantum yield approaches 20%, which is a higher value than one determined for redaporfin (Φ_F_ = 0.138).^49^ Notably, the electronic absorption spectrum shows several options for excitation–in the Q_*x*_ band −515 nm and in the red part 680–690 nm (Figure 1, λ_Exc1_ and λ_Exc2_, respectively). The second excitation wavelength is situated in the middle of the optical window, making this bacteriochlorin suitable for real-time in vivo imaging as the photons in this range can penetrate deeper into the tissues, and this wavelength does not coincide with the fluorescence.

The properties of photosensitizers triplet states are critical for their success in PDT. Charge transfer from the photosensitizer to molecular oxygen with free radical formation (Type I process), or energy transfer to generate singlet oxygen (Type II process), originates from its triplet excited state. Long-lived triplet states with tens of microsecond lifetimes are required for efficient interaction with molecular oxygen in neoplastic tissues, where molecular oxygen concentrations can be 2 orders of magnitude lower than in organic solvents. We investigated the triplet states of the photosensitizers in aerated and nitrogen-saturated toluene solutions and found that they are adequately fitted by monoexponential decays (Figures S5 and S6, Supporting Information). The triplet lifetimes obtained from such decays are presented in Table 1. The transient decay times when oxygen is present are unaffected by the observed wavelength and display typical behavior of triplet states transferring energy quantitatively to oxygen molecules. The lifetimes of the triplet states for **F**_**4**_**TPP-MS** closely resemble those reported for halogenated porphyrins, while for **F**_**4**_**BMet**, they are even longer than for halogenated bacteriochlorins previously used in various in vivo models and clinical studies.^3,36,41^ Moreover, the triplet lifetimes are remarkably longer in nitrogen-saturated solutions and are probably limited by the residual oxygen present in the solutions. For **F**_**4**_**TPP-MS**, lifetimes increased from 590 ns under oxygen conditions to 45 μs after nitrogen saturation of the solvent, and for **F**_**4**_**BMet**, they increased from 270 ns to 28 μs. The shorter triplet lifetimes of the porphyrins **F**_**4**_**TPP-MS** and **F**_**4**_**PMet** in aerated toluene are consistent with diffusion-controlled oxygen quenching of the triplet states ([O_2_] (^3^Σ^–^_*g*_) = 2 mM, *k*_q_ ≈ 8 × 10^8^ M^–1^ s^–1^). Compared with the porphyrins in aerated solutions, **F**_**4**_**BMet** presents a shorter triplet state lifetime, which suggests a charge transfer interaction with molecular oxygen.

A photosensitizer can be regarded as a very special type of catalyst that maintains the rate of a chemical transformation (e.g., triplet oxygen → singlet oxygen) as long as it absorbs photons and is not photobleached. The photodecomposition quantum yield (Φ_PD_) is defined as the ratio between the initial rate of disappearance of the photosensitizer molecule and the initial rate of absorption of photons being a good measure of the ability of the photosensitizer molecules to generate ROS before bleaching. Table 1 presents Φ_PD_ values obtained for the photosensitizers used in this work. The presence of secondary sulfonamides (Ph-NHSO_2_R) presents substantially lower values (Φ_PD_ < 4 × 10^–5^), adequate for PDT. Interestingly, the photostability of **F**_**4**_**BMet** (Φ_PD_ = 2 × 10^–5^) is improved compared to other sulfonamide bacteriochlorins.^36^ This phenomenon is elucidated by theoretical calculations presented in the work. Lowering the HOMO energy hinders the oxidation of the ring while reducing the LUMO energy facilitates reduction more effectively in **F**_**4**_**BMet** than in **H**_**4**_**BMet** (Figures S2 and S3). Consequently, fluorination remarkably enhances the stability of the bacteriochlorins.

Looking for a photosensitizer characterized by high singlet oxygen quantum yields and adequate photostability, we selected three photosensitizers, **F**_**4**_**TPP-MS**, **F**_**4**_**PMet,** and **F**_**4**_**BMet**, to expand our studies. However, for fluorescence imaging, only one of those three compounds reached the appropriate fluorescence quantum yield to be considered a potential fluorescence imaging agent – **F**_**4**_**BMet** with Φ_F_ = 0.20. This quantum yield is very similar to that obtained for silicone benzoxyphtalocyanine which shows great potential as a fluorescence agent in real-time imaging, with high selective accumulation in the tumor.^34^

### Detection of Reactive Oxygen Species

**Figure 2:**
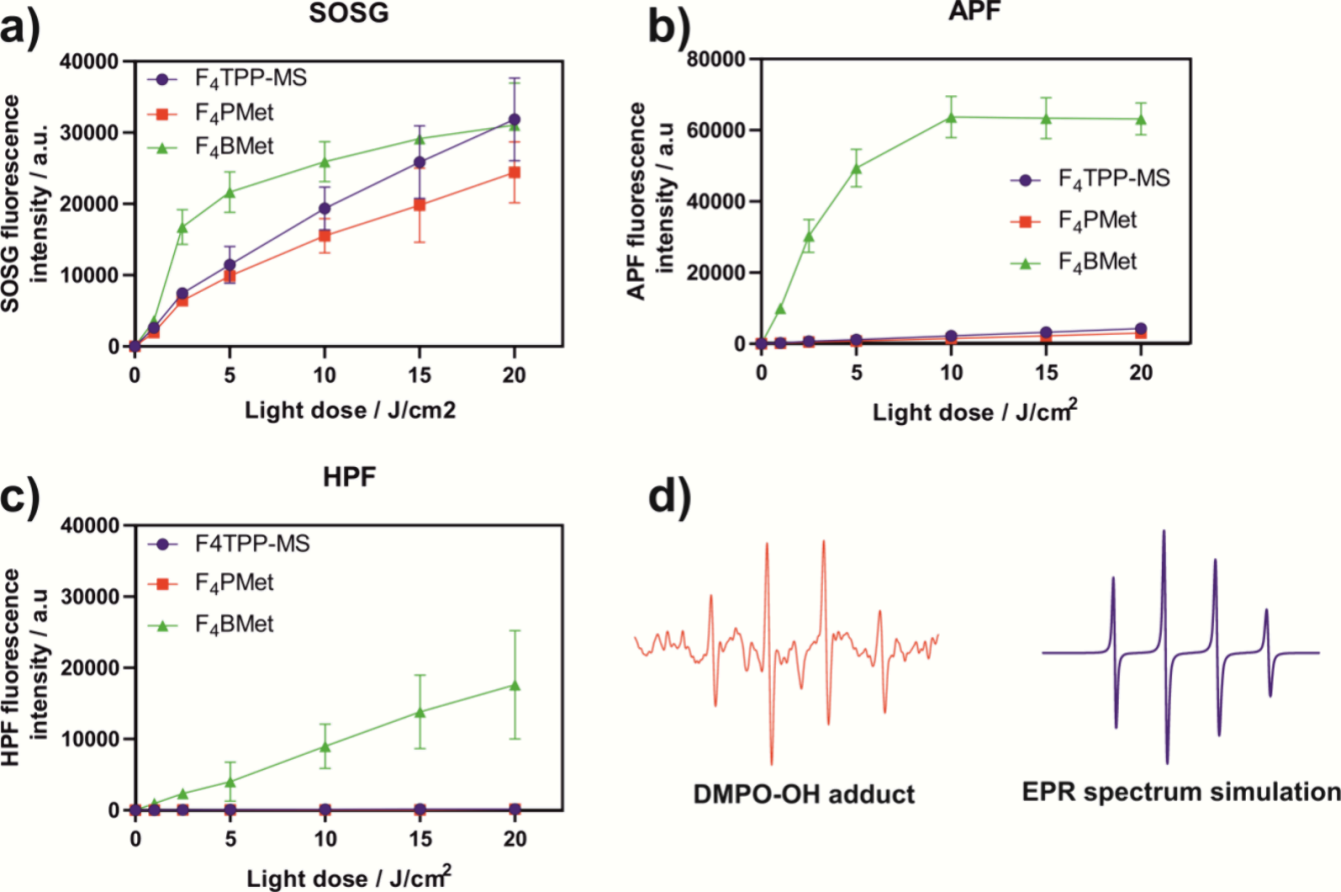
Green fluorescence of ROS probes– (a) SOSG, (b) APF, and (c) HPF after irradiation of 5 μM aqueous solution (PBS, 0.5% DMSO) of each photosensitizer with red (635 ± 20 nm) for porphyrins (blue, red) or NIR light (735 ± 20 nm) for bacteriochlorin (green), respectively. Results are the average of at least three experiments ± SEM. (d) EPR spectrum of hydroxyl radical-DMPO adduct observed after irradiation of **F**_**4**_**BMet** aqueous solution with NIR light along with DMPO–OH computer simulated spectrum.

The singlet oxygen quantum yields (Φ_Δ_) of the porphyrins studied in this work are quite high, in the 0.59–0.74 range.^47^ The corresponding value for **F**_**4**_**BMet**, Φ_Δ_ = 0.36, was obtained from the pulsed laser energy dependence of the time-resolved singlet oxygen emission intensity at 1270 nm using phenalenone as reference.^50,51^ Singlet Oxygen Sensor Green (SOSG) is a highly selective singlet oxygen probe, with low sensitivity for superoxide ions and hydroxyl radicals. 3′-(*p*-aminophenyl) fluorescein (APF) and 3′-(p-hydroxyphenyl) fluorescein (HPF) are known to be very sensitive to hydroxyl radicals but not to singlet oxygen. Once the radicals are generated, the two probes react with them to form a well-known fluorophore - fluorescein. The use of these probes gives insight into the nature of the generated ROS. As shown in Figure 2a, porphyrins - **F**_**4**_**TPP-MS**, **F**_**4**_**PMet**, and bacteriochlorin **F**_**4**_**BMet** generate comparable amounts of green fluorescence after irradiation at 635 ± 20 nm (porphyrins) or at 735 ± 20 nm (bacteriochlorin) with SOSG, consistent with their Φ_Δ_. However, **F**_**4**_**BMet** generates more singlet oxygen in a shorter time compared to both porphyrins. The fluorescence intensity of fluorescein generated by APF and HPF probes after irradiation is 1 order of magnitude higher for the bacteriochlorin than for the porphyrins. This is clear evidence that hydroxyl radicals are generated after bacteriochlorin irradiation, possibly in reactions subsequent to the generation of superoxide ions. It is worth noting, however, that the apparent plateau in the fluorescein signal for APF is due to the limit of detection rather than reduced ROS generation. This is confirmed by fluorescence measurements with HPF, where the signal shows a linear increase in fluorescence through all the light doses, which underlines the ability of bacteriochlorin to generate hydroxyl radicals. In comparison, HPF fluorescence after porphyrins irradiation showed little to no generation of hydroxyl radical. The ability of bacteriochlorins to participate extensively in both Type I and Type II processes has been demonstrated and their increased PDT activity has been associated with Type I photochemistry.^52,53^ Photosensitizers that generate ROS by Type I and Type II photochemical reaction may be more useful in the clinic for tumors characterized by hypoxic microenvironment because the generation of radicals by Type I reactions may not require oxygen.^52−54^ The different fluorescence intensities registered with the APF and HPF probes are related to their intrinsic response to hydroxyl radicals. The generation of the hydroxyl radical was further confirmed by electron paramagnetic resonance spin trapping. The illumination of **F**_**4**_**BMet** (5 μM) aqueous solution (PBS, 0.5% DMSO) in the presence of 5,5-dimethyl-1-pyrroline-N-oxide (DMPO, 50 mM), a known spin trap of hydroxyl radical, led to an EPR spectrum (Figure 2d, left). The line shape and the hyperfine (*h*_f_) splitting of the signal are typical of the spin adduct formed between DMPO and the hydroxyl radical, DMPO–OH (*a*_N_ = 14.9 G, *a*_Hβ_ = 14.9 G, *g* = 2). This spin adduct was not observed in the absence of light or in a nitrogen-saturated solution. Figure 2d (right) shows a simulation of the DMPO–OH adduct.

### Time-Dependent Cellular Uptake

**Figure 3:**
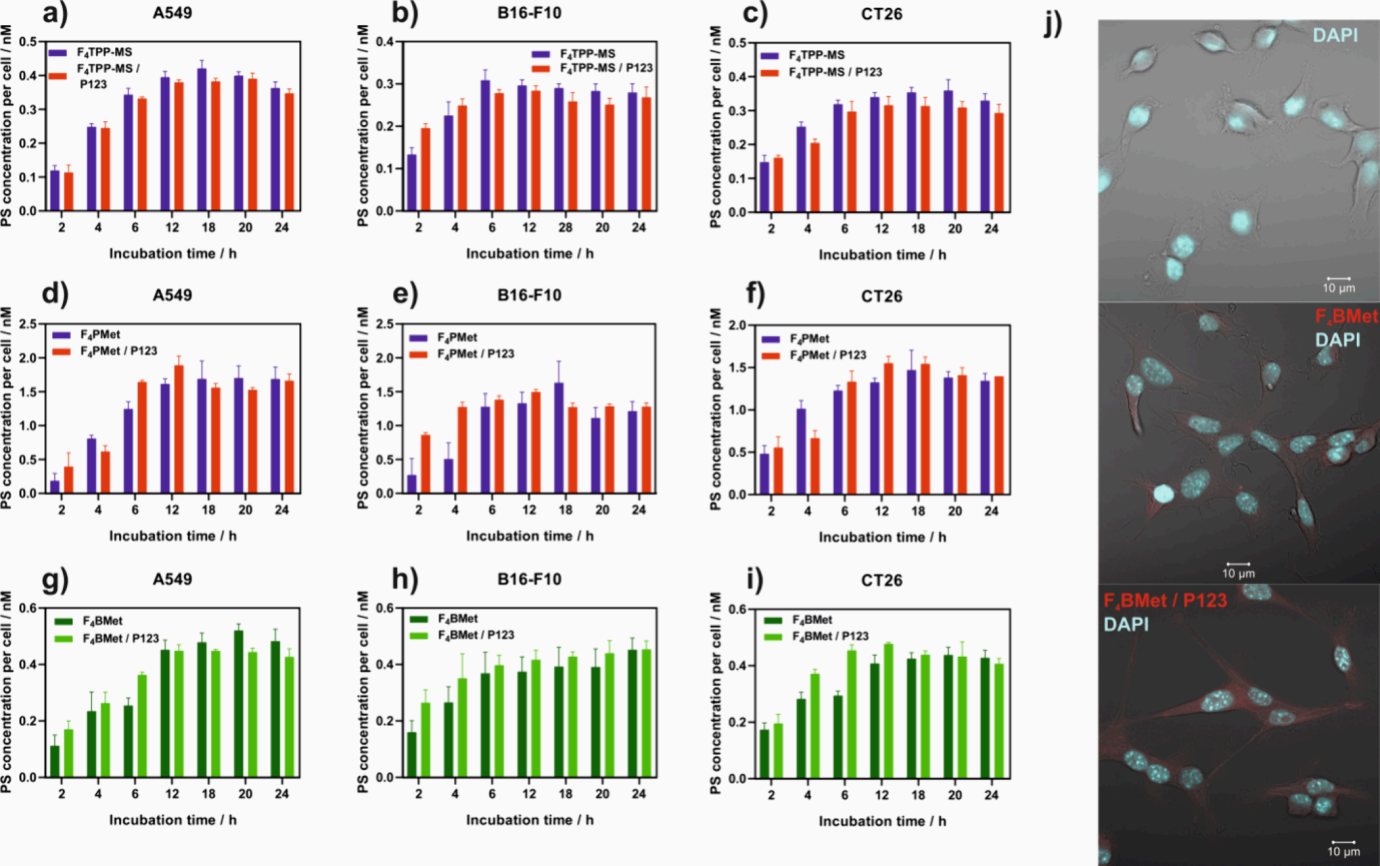
(a–i) Time-dependent uptake of **F**_**4**_**TPP-MS**, **F**_**4**_**PMet,** and **F**_**4**_**BMet** prepared in PBS/0.5% DMSO or encapsulated in P123 micelles by cancer cells measured by fluorescence intensity. Results are the average of at least three experiments ± SEM. (j) Fluorescence confocal imaging of CT26 cells (upper–control, middle–after 24 h incubation with **F**_**4**_**BMet** dissolved in 1% DMSO in PBS, lower–after 24 h incubation with **F**_**4**_**BMet/P123**).

Several nanostructured vehicles can not only serve as carriers for drug delivery but also modulate the biological response and photochemical properties of delivered drugs. It has been argued that photosensitizer-Pluronic materials may result in both passive targeting of the tumor cells by the EPR effect and modulation of tumor drug resistance.^55−58^ It is known that P123 micelles are efficiently internalized by endocytosis. This motivated the use of sulfonamide porphyrins and bacteriochlorin formulations with these block copolymers. The particle sizes of **F**_**4**_**TPP-MS**, **F**_**4**_**PMet,** and **F**_**4**_**BMet** after encapsulation in polymeric micelles were controlled by dynamic light scattering (DLS), and for each photosensitizer, the average size reached about 24–28 nm (Figure S7, Supporting Information). The time-dependent cellular uptake of **F**_**4**_**TPP-MS**, **F**_**4**_**PMet,** and **F**_**4**_**BMet** was investigated in A549, B16–F10, and CT26 cells after exposure to 20 μM (porphyrins) or 5 μM (bacteriochlorin) photosensitizer solutions (Figure 3a–i). The uptake of the free-form of photosensitizers was compared to the uptake of the micellar formulation. All tested compounds reached the accumulation plateau in all tested cancer cell lines after 12–18 h. Although exposed to similar concentrations, **F**_**4**_**PMet** tends to accumulate 3–5 times more effectively than **F**_**4**_**TPP-MS**. Encapsulation in P123 micelles gave mixed results. **F**_**4**_**TPP-MS/P123** showed a decreased uptake in comparison to the free form of the photosensitizer in all tested cell lines. **F**_**4**_**PMet** gave different results, as the uptake of **F**_**4**_**PMet/P123** is faster, especially in B16–F10 cells, and reaches a plateau after 6 h of incubation. **F**_**4**_**BMet** shows the fastest uptake in CT26 and B16–F10 cells, and the encapsulation in micelles shortens the time of maximum accumulation from 12 to 6 h in both cell lines. For A549 initially, micellized **F**_**4**_**BMet** accumulates faster and reaches the plateau after 12 h; however, after 12 h, the free-form **F**_**4**_**BMet** accumulation still increases and reaches a maximum at around 20 h of incubation.

Confocal microscopy of **F**_**4**_**BMet** in CT26 cells after 24 h incubation with 15 μM (Figure 3j) in two formulations −1% DMSO/PBS and P123 micelles revealed accumulation of the compound in several organelles. **F**_**4**_**BMet** was excited at 405 nm with emission observed over 745 nm wavelength, and the DAPI used for nucleus staining was excited with 355 nm laser. Additional images taken after incubation with different **F**_**4**_**BMet** concentrations are presented in Figure S8, Supporting Information.

### Cytotoxicity in Dark and Photodynamic Effect

**Figure 4:**
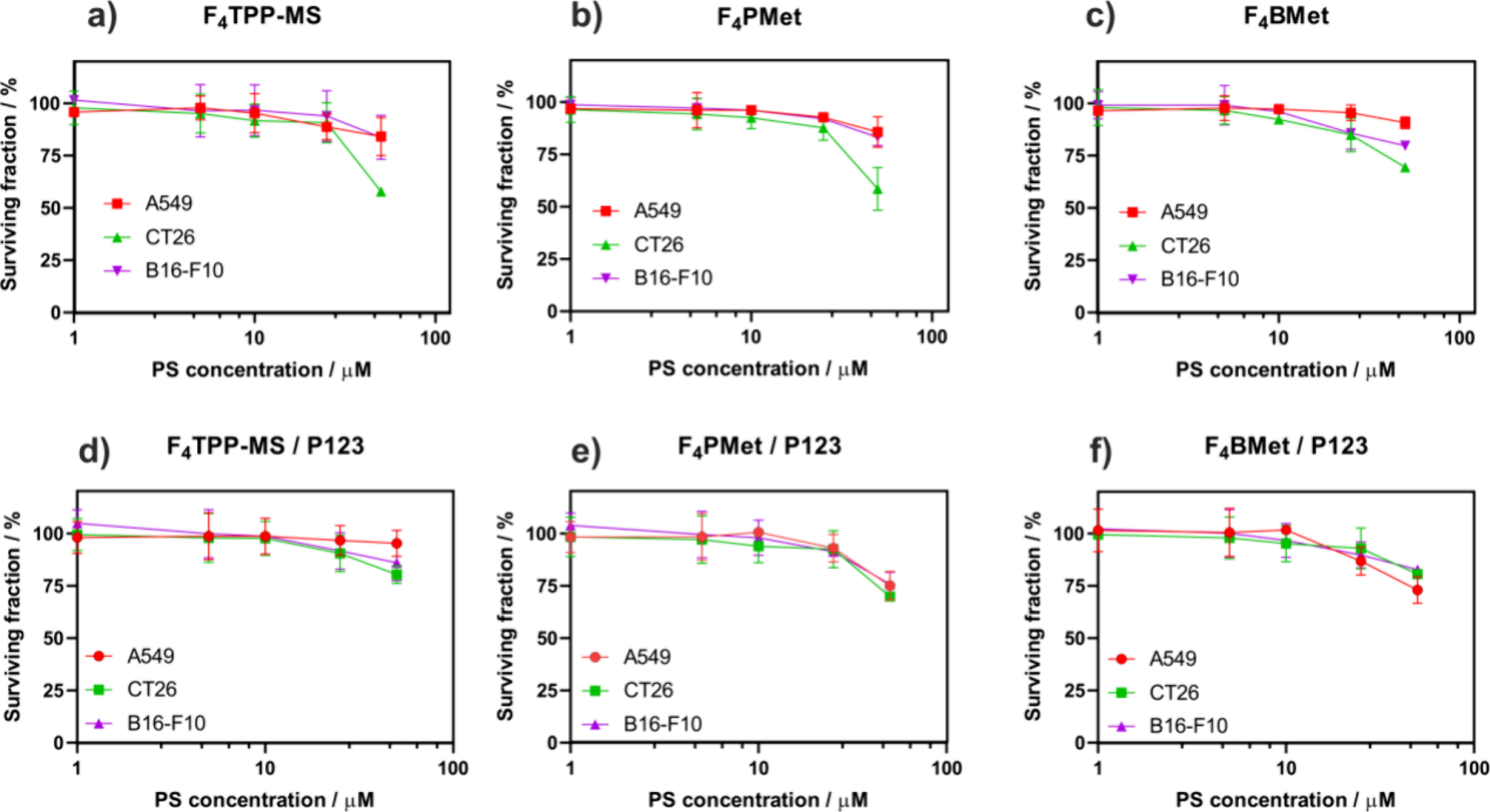
Cytotoxicity against several cancer cell lines without the irradiation of (a) **F**_**4**_**TPP-MS**, (b) **F**_**4**_**PMet**, (c) **F**_**4**_**BMet,** and (d) **F**_**4**_**TPP-MS/P123,** (e) **F**_**4**_**PMet/P123,** and (f) **F**_**4**_**BMet/P123** in the concentration range (1–50 μM). Results are the average of at least three experiments ± SEM.

Low dark cytotoxicity is one of the prerequisites for photosensitizers in PDT and fluorescence imaging. This evaluation allows to establish a concentration that is safe to use without any adverse effects and, therefore, to choose a proper concentration for further examination of the photodynamic effect. The subsequent toxicity and photodynamic effect studies were carried out after incubation for 18–20 h against A549, CT26, and B16–F10 cells. The cell viability was assessed using the AlamarBlue assay. Figure 4 shows that the photosensitizers have a negligible effect on the cell viability within the concentrations range from 1–25 μM. However, there is a dose-dependent increase in the cytotoxicity for concentrations larger than 25 μM. All the IC_50_ values measured are >50 μM. Encapsulation of all three tested photosensitizers in Pluronic P123 micelles resulted in the reduction of dark cytotoxicity in the range of 1–50 μM.

The photodynamic effect was assessed as the ability of the tested compounds to generate ROS that kill various cancer cells: A549, CT26, and B16–F10. The cell morphology before and after the PDT was also examined by optical microscopy (Figure S9, Supporting Information). The concentrations employed were selected to be above the 90% survival fraction in the dark (Figure 4) after 18–20 h of incubation (20 μM for porphyrins and 5 μM for bacteriochlorin).

**Figure 5:**
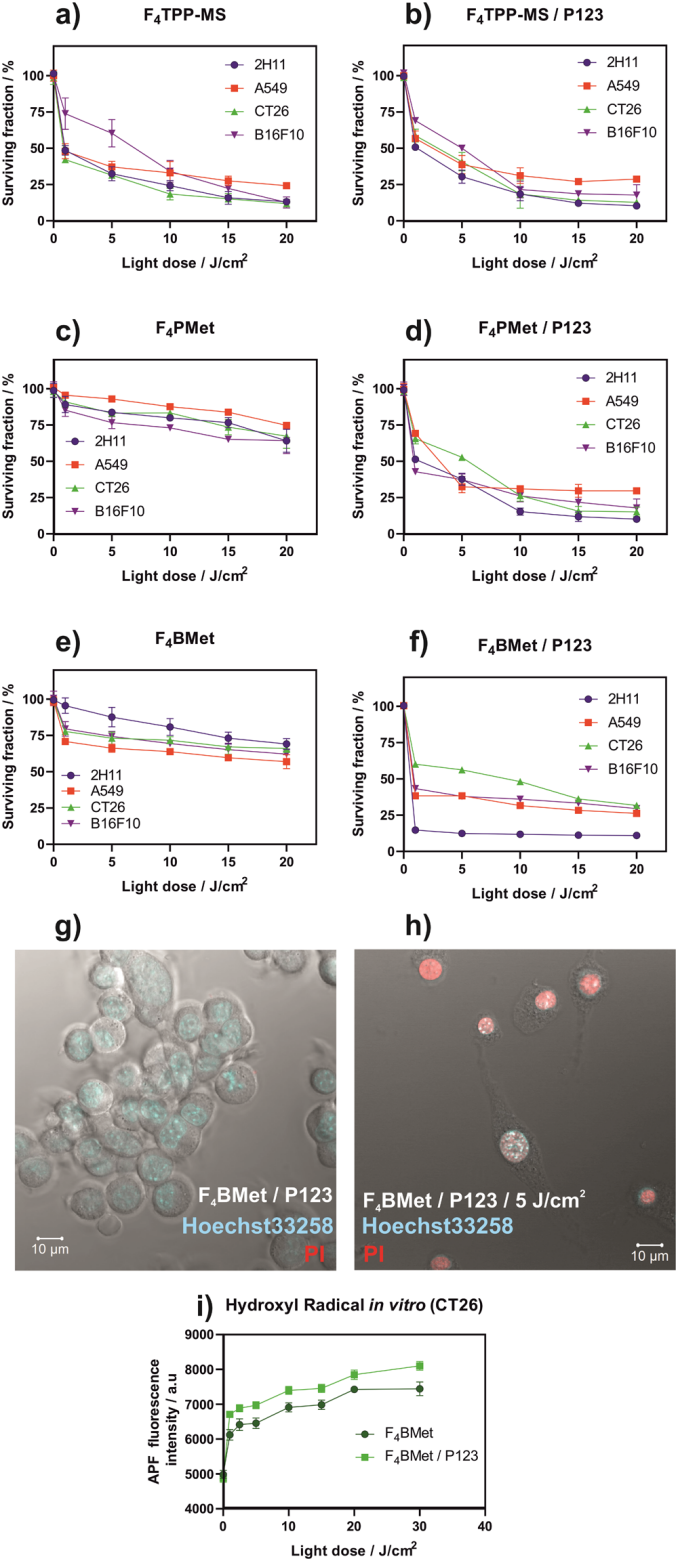
Photodynamic effect against several cancer cells of **F**_**4**_**TPP-MS** (a, b) or **F**_**4**_**PMet** (c, d) and **F**_**4**_**BMet** (e, f) performed after optimal incubation time (determined experimentally) with 20 μM (for porphyrins) or 5 μM (for bacteriochlorin) photosensitizer solution (PBS, 0.5% DMSO) or in P123 micelles and irradiation with red (635 ± 20 nm) for porphyrins or NIR light (735 ± 20 nm) for bacteriochlorin, respectively. (g, h) Confocal fluorescence imaging of the **F**_**4**_**BMet**/P123 photodynamic effect against CT26 cells (before and after 5 J/cm^2^ of irradiation with NIR light). (i) In vitro generation of hydroxyl radical in CT26 cells was evaluated for **F**_**4**_**BMet** (5 μM) in two formulations after NIR (735 ± 20 nm) irradiation. Results are the average of at least three experiments ± SEM.

**F**_**4**_**TPP-MS** in free form appears to be more effective in the destruction of various cancer cells compared to **F**_**4**_**PMet**, despite the latter higher uptake and accumulation in the cells. After encapsulation in Pluronic P123 micelles, both porphyrins have similar effectiveness. However, encapsulation of **F**_**4**_**TPP-MS** does not change the effectiveness of cancer cell destruction. These results suggest that micelles do not necessarily increase the bioavailability of porphyrins but probably protect the compounds against oxidation. **F**_**4**_**BMet**, similarly to its porphyrin analogue, increases its effectiveness after encapsulation. The **F**_**4**_**BMet/P123** result shows that despite exposure to a lower concentration of PS (5 μM), it possesses the highest effectiveness against all cancer cell lines tested, with the highest effectiveness against colon cancer cells. Even after illumination with a light dose of 1 J/cm^2^, almost 80% of cancer cells were destroyed. This suggests that the Pluronic P123 formulation protects **F**_**4**_**BMet** from bleaching and degradation in biological media and may favor more lethal subcellular localizations. Fluorescence confocal imaging of live/dead cells (Figures 5g,h and S10 in the Supporting Information) showed the destruction of cancer cells after incubation of CT26 cells with **F**_**4**_**BMet**/P123 and irradiation with NIR light. The dead or damaged cells were stained with propidium iodide (PI) which binds specifically to the DNA of damaged cells. Before irradiation, no red fluorescence was detected, indicating no toxicity toward cancer cells. After irradiation with a light dose of 5 J/cm^2^, clear changes in morphology are observed in the bright field confocal imaging, and clear red fluorescence from PI bonded to damaged cells is visible.

As the detection of hydroxyl radical in vitro suggests (Figure 5i), **F**_**4**_**BMet** shows an enhanced production of this type of ROS after encapsulation in micelles, which further underlines the theory of protection against degradation. The bacteriochlorin **F**_**4**_**BMet** is especially phototoxic toward a variety of cancer cells, especially toward CT26 cells, and their biocompatible characteristics open doors for new ways of targeting. It is worth noting, that bacteriochlorin achieved similar (for A549 and B16–F10 cells) or even better results (CT26 cell line), compared to porphyrins, with four times lower concentration used. As **F**_**4**_**BMet** was effective even at lower concentrations, it was selected for further evaluation of biological properties in a 3D in vitro model of hiPSC-derived colonic organoids and in vivo experiments.

### pH-Dependent Properties of F_4_BMet

**Figure 6:**
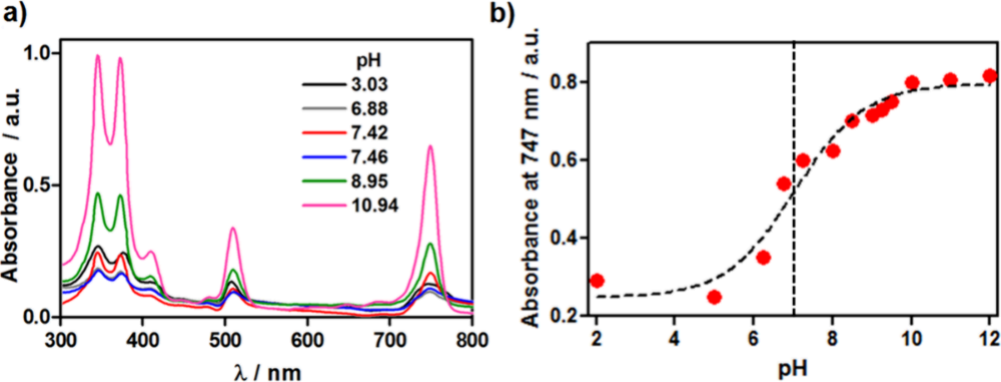
Environment influence on the aggregation of **F**_**4**_**BMet.** (a) Effect of pH on the absorption of **F**_**4**_**BMet** and (b) p*K*_a_ determination based on changes in absorbance at 747 nm.

One of the most characteristic properties of tumors is fast growth, which limits the availability of oxygen. Through reduction of the blood supply and the creation of hypoxic conditions, the TME may become moderately acidic (pH 5.5–6.5).^59^ Therefore, it is crucial to establish how a photosensitizer behaves not only under physiological conditions (pH 7.4) but also under conditions of TME and tailor the delivery system of the photosensitizer to enhance the selectivity of the compound. Therefore, the effect of pH on the electronic absorption spectra of **F**_**4**_**BMet** was investigated. Figure 6a shows the titration of **F**_**4**_**BMet** starting from an aqueous solution with a pH of 11.64. The absorption changes are consistent with aggregation in an acidic solution and an acid dissociation constant p*K*_a_ = 6.98 of **F**_**4**_**BMet** (Figure 6b). This is significantly lower than the p*K*_a_ value of CH_3_SO_2_NHPh, 12.9. Fluorination of the aromatic ring increases the acidity of the sulfonamide, as expected.^60^

The pH-dependence of **F**_**4**_**BMet** offers the possibility to control its internalization in tumor cells and, therefore, can enhance PDT efficacy. Photosensitizer aggregation reduces triplet state lifetime and ROS yield, thereby adversely affecting efficacy. Considering the pH-dependent aggregation of **F**_**4**_**BMet**, we employed two formulations for an in vivo evaluation. One of them is based on Pluronic P123 micelles developed in our group recently,^46^ and the other one is a glycylglycine buffer (GlyGly, pH 8.6) that allows for solubilization of this bacteriochlorin without any signs of aggregation.

### hiPSC-Derived Colonic Cancer Organoids

Legislative advances removed the obligation to conduct research using animals to obtain FDA approval because biological engineering with organs-on-chip and organoids now offers realistic models.^61^ It seems reasonable, therefore, to include these models in preclinical studies, even if it is still risky to completely replace lab-animal testing. Nevertheless, by using organoids in preclinical studies, we may reduce the use of animals in research in comparison with studies conducted in the past.^7^ In this work, the human colorectal cancer (CRC) organoid model was developed with the preservation of the digestive system’s physiological features. We evaluated the uptake, dark cytotoxicity, and photodynamic effect of **F**_**4**_**BMet** in two different formulations (Pluronic P123-based micelles and GlyGly buffer, as for in vivo studies) against CRC organoids.

### Uptake

**Figure 7:**
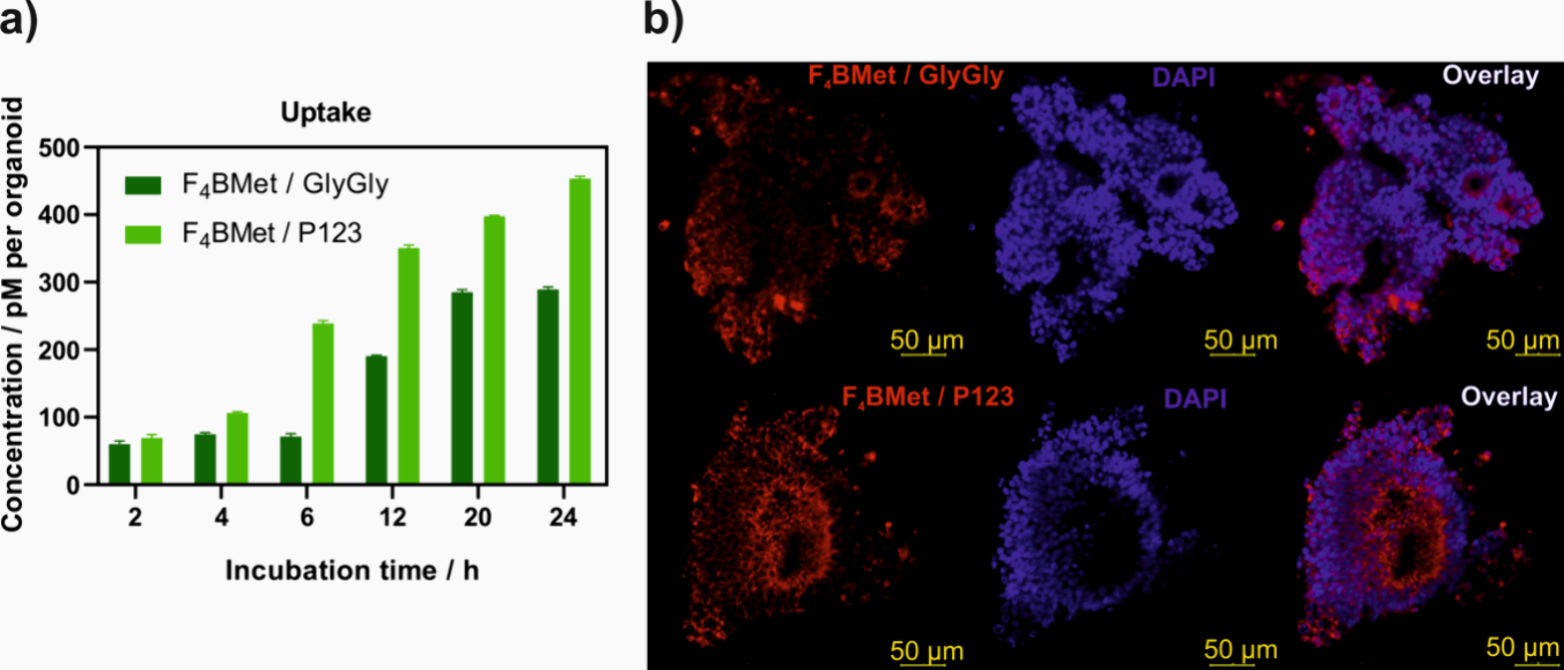
(a) Time-dependent uptake of **F**_**4**_**BMet** prepared in GlyGly buffer and encapsulated in P123 micelles by hiPSC-derived CRC organoids, measured by fluorescence intensity. Results are the average of at least three experiments ± SEM. (b) Fluorescence confocal images of CRC organoids after 24 h of incubation with **F**_**4**_**BMet** in GlyGly buffer or P123 micelles.

**F**_**4**_**BMet** uptake by CRC organoids was compared for both formulations (Figure 7a). hiPSC organoids were treated with a 2 mg/kg BW concentration of the photosensitizer. **F**_**4**_**BMet/P123** shows a higher and faster accumulation into the organoid structure compared to **F**_**4**_**BMet/GlyGly**. After 6 h of incubation, there is a visible difference in the accumulation of the photosensitizer (almost 2-fold higher concentration for **F**_**4**_**BMet/P123** formulation). After 24 h, **F**_**4**_**BMet/P123** formulation reached an approximate concentration of 450 pM when **F**_**4**_**BMet/GlyGly** was about 300 pM.

Fluorescence confocal imaging shows the differences in **F**_**4**_**BMet** localization between the two formulations (Figure 7b). After 24 h of incubation, **F**_**4**_**BMet/P123** appears to penetrate deeper into the organoid structure, compared to **F**_**4**_**BMet/GlyGly**, which accumulates mainly at the periphery of the structure. It can be speculated that in the Pluronic-based formulation **F**_**4**_**BMet/P123** undergoes a more uniform distribution in the organoid area in comparison to **F**_**4**_**BMet** solubilized with GlyGly buffer. It is possible that pH changes cause destabilization of the **F**_**4**_**BMet/GlyGly** buffered solution, causing aggregation of the photosensitizer in the peripheric areas of the organoid, limiting its availability in the deeper regions.

### Cytotoxicity in the Dark

**Figure 8:**
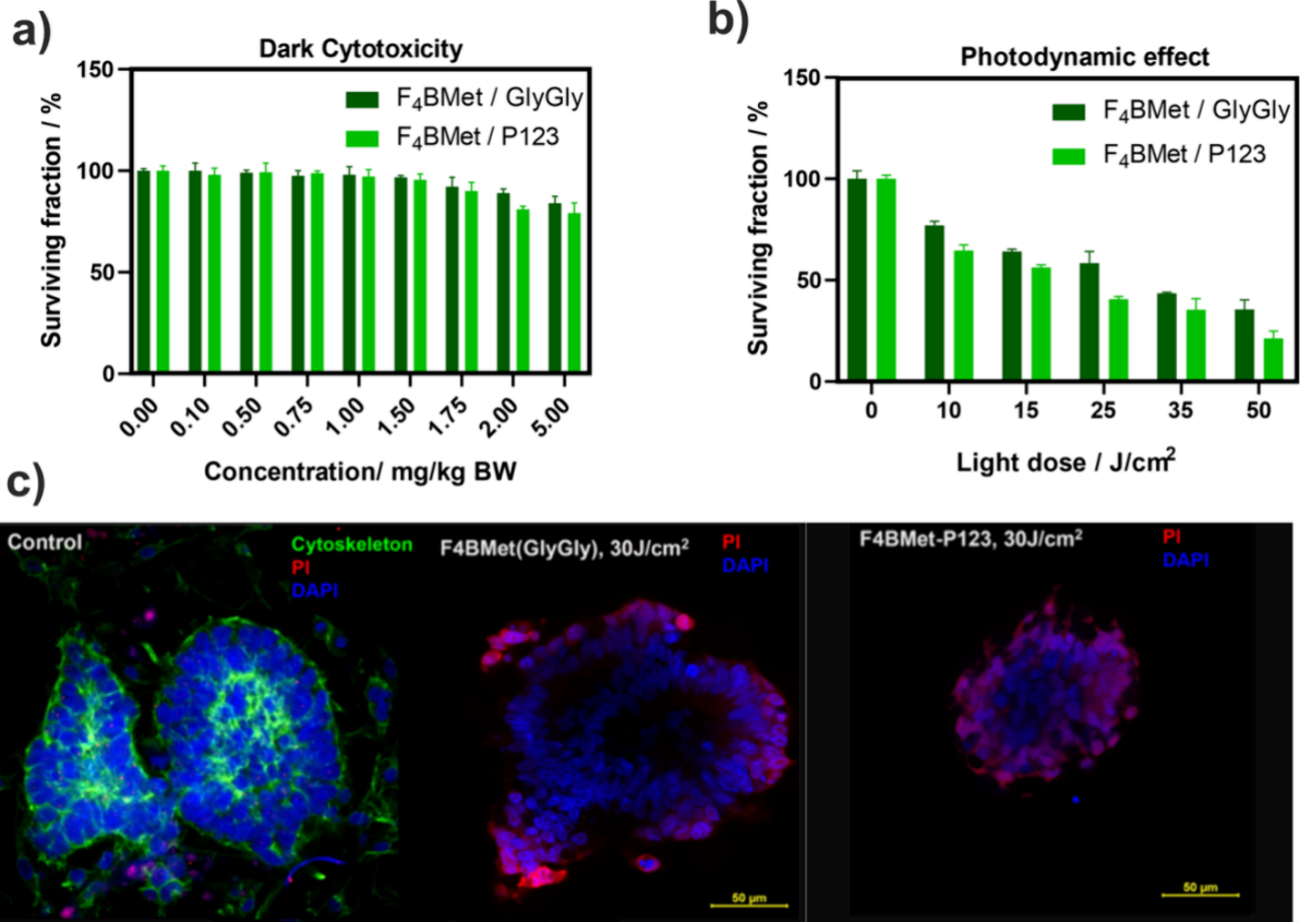
(a) Dark cytotoxicity of **F**_**4**_**BMet** in P123 micelles and GlyGly buffer investigated against hiPSC-derived CRC organoids. (b) Photodynamic effect against organoids of **F**_**4**_**BMet** in two formulations–P123 micelles and GlyGly buffer, performed after 24 h incubation time with 4 μM photosensitizer without (PBS, 0.5% DMSO) and irradiation with NIR light (735 ± 20 nm). Results are the average of at least three experiments ± SEM. (c) Confocal images of live/death staining with DAPI and PI after 24 h incubation of CRC organoids with **F**_**4**_**BMet** in P123 micelles and GlyGly buffer and irradiation with 30 J/cm^2^ (735 ± 20 nm).

To further evaluate safety, a dark cytotoxicity assay was performed on CRC organoids with different concentrations of **F**_**4**_**BMet**. The results suggest that the compound remains relatively nontoxic in the absence of light, over a wide range of concentrations tested (Figure 8a). A slight decrease in the viability of organoids can be observed once the concentration exceeds 1.75 mg/kg BW and is more visible for **F**_**4**_**BMet** encapsulated in P123 than in the GlyGly buffer. However, even at a concentration of 5 mg/kg, the surviving fraction was almost 80% with **F**_**4**_**BMet/P123** and 84% with **F**_**4**_**BMet/GlyGly**.

### Photodynamic Effect

The photodynamic effect was compared for the two **F**_**4**_**BMet** formulations against CRC organoids, applying different light doses (0–50 J/cm^2^) at 735 ± 20 nm wavelength. In both cases, a concentration of 2 mg/kg was used. At all light doses, **F**_**4**_**BMet/P123** showed a photodynamic potential slightly higher than that of **F**_**4**_**BMet/GlyGly**. At a maximum light dose of 50 J/cm^2^, **F**_**4**_**BMet/P123** succeeded in killing 79% of cells compared to **F**_**4**_**BMet/GlyGly** which resulted in 65% of destroyed cells within the structure (Figure 8b). **F**_**4**_**BMet/P123** higher effectiveness can be attributed to its higher uptake into the organoid structure and similarly to 2D cell culture, protection from oxidative degradation. Fluorescence confocal microscopy images (Figure 8c) confirm the results of the cell viability analysis after PDT. Staining of living and dead cells with propidium iodide (PI) after PDT with a dose of 30 J/cm^2^ (735 ± 20 nm) showed a significant reduction in the size of the organoid structure. Staining with PI which binds to the DNA of dead and/or damaged cells shows the damage to the cells even deeply inside the organoid structure after incubation with **F**_**4**_**BMet/P123**. The weaker effect of **F**_**4**_**BMet/GlyGly** may be due to its limited penetration of the CRC organoid structure. PI stained only the peripheric cells of the structure similar to Figure 7b, whereas for **F**_**4**_**BMet/P123**, PI stained almost all remaining cells in the structure of the organoid.

### In Vivo Fluorescence Detection of F_4_BMet

**Figure 9:**
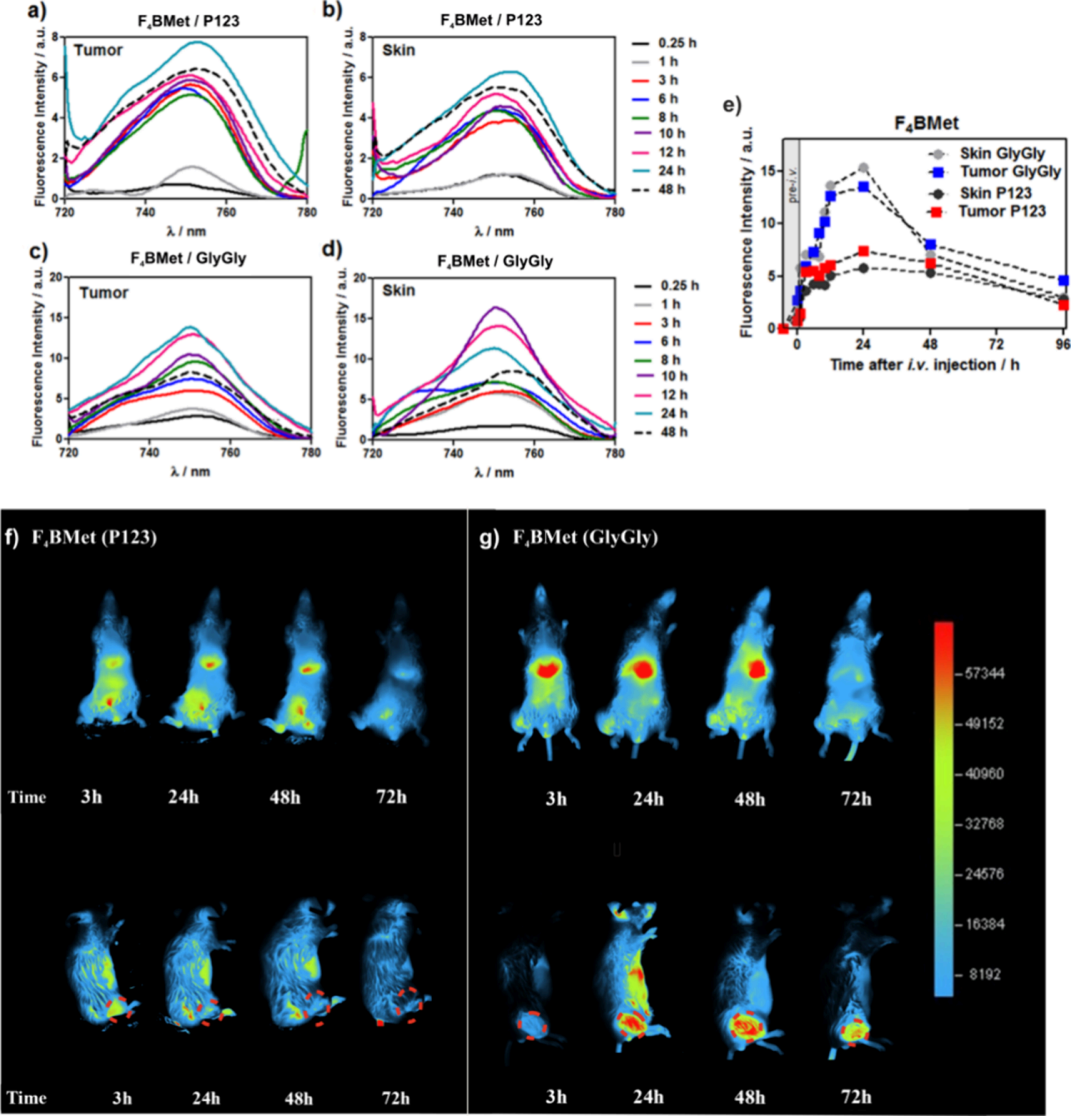
Fluorescence spectra of **F**_**4**_**BMet** prepared in P123 micelles (a, b) and GlyGly buffer (c, d) registered from tumor and skin areas at various postinjection times using fiber optics (λ_ex_ = 510 nm, λ_em_ = 720–780 nm). (e) Kinetics of **F**_**4**_**BMet** accumulation in skin and CT26 tumor with a comparison of both applied formulations of photosensitizer (GlyGly buffer, P123 micelles). (f, g) Whole-body fluorescence imaging of BALB/c mice bearing CT26 tumor in the right leg, collected after 3, 24, 48, and 72 h after the *i.v.* administration of 1.5 mg/kg of **F**_**4**_**BMet** with excitation at 680 nm and fluorescence emission at 740–780 nm. The tumor area was indicated with a red dashed circle.

The outstanding results of in vitro studies encouraged us to evaluate the efficacy of **F**_**4**_**BMet** in CT26 tumor-bearing BALB/c mice after intravenous administration of the photosensitizer. This cell line was chosen because our 3D in vitro experiments were conducted on the model of human colon cancer. First, we determined the possibility of accumulation of **F**_**4**_**BMet** in tumors using noninvasive fluorescence imaging. Fluorescence measurements were used to monitor the possibility of bacteriochlorin accumulation in the tumor and skin up to 96 h post-injection (*i.v*.). The fluorescence spectra of **F**_**4**_**BMet** were registered using optical fiber at different times post-injection. As shown in Figure 9a–d, there is no red fluorescence in the tumor area at pre-*i.v*. time (background). Immediately after *i.v*. injection, a red fluorescence signal specific for bacteriochlorin was observed in the tumor area. In general, this signal increases over the first 24 h. Nevertheless, fluorescence signals can also be observed in healthy tissue areas (skin), which indicates that some amount of bacteriochlorin accumulated in the skin, especially for GlyGly formulation. The uptake of **F**_**4**_**BMet/GlyGly** by skin is faster and reaches its maximum 10 h post-injection. P123 presents an enhanced accumulation in tumor tissue, with a maximum at 24 h that is higher than that in skin. The optimal uptake at 24 h was also observed in the case of **F**_**4**_**BMet/GlyGly**, but for this formulation, we did not observe significant differences in photosensitizer accumulation in tumor vs skin, which can suggest lower selectivity against cancer cells.

Fluorescence imaging of mice with CT26-tumors in the right leg after injection of **F**_**4**_**BMet** delivered in P123 and GlyGly (Figure 9f,g left and right panel, respectively) shows that **F**_**4**_**BMet/GlyGly** exhibits higher accumulation in the tumor area, compared to **F**_**4**_**BMet/P123**, and reaches a maximum after 24 h post-injection. The concentration in the tumor remains high after 48 h, and the photosensitizer is still detectable 72 h post-injection. However, after dissolving in buffer solution, **F**_**4**_**BMet** seems to accumulate in the liver and remains there for 48 h. This effect was also observed in **F**_**4**_**BMet/P123;** however, it possesses significantly decreased accumulation in the liver. Clearly, **F**_**4**_**BMet** accumulates in the tumor, which makes it a potential agent for diagnostics and image-guided PDT.

These results confirmed that **F**_**4**_**BMet** localizes in tumor tissue with optimal time ca. 24 h after administration, which indicates that this is the appropriate drug-to-light interval (DLI) for photodynamic treatment. Moreover, in both cases, background fluorescence intensity decreases over time and nearly disappears 96 h post-injection (Figure 9e). The observed differences in the accumulation of PS in dependence on its formulation may be related to the application of P123 micelles that may significantly prolong the circulation time of PS and favor selective targeting of tumor tissue and TME via the EPR effect.^46^

The results described in this section point to another key issue regarding the design of effective PDT protocols. For the majority of photosensitizers used in PDT, photons from the blue or green ranges are used to study their biodistribution or detection. These wavelengths have the potential to excite other pigments such as melanin, hemoglobin, vitamin B12, and bilirubin, leading to disruptions and possibly inflated findings. The unique optical properties of **F**_**4**_**BMet** allow in vivo fluorescence imaging of the whole mouse using a 680 nm laser. These photons do not excite the already-mentioned endogenous compounds and can penetrate deeper into the tissue.

### Photodynamic Therapy

**Figure 10:**
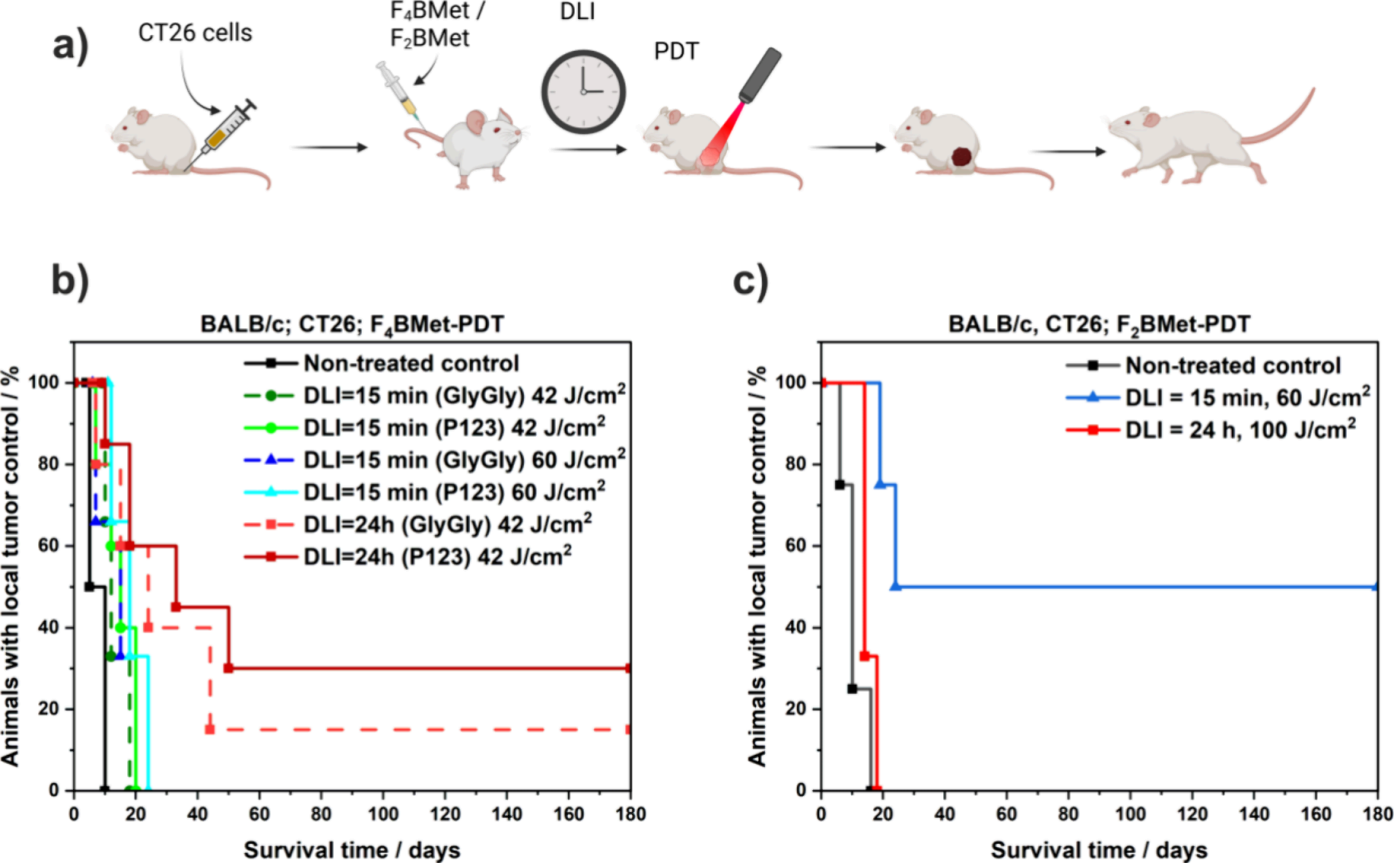
(a) Schematic of in vivo photodynamic effect experiment. CT26 cells are inoculated into the right leg of BALB/c mice. When the tumors reach an appropriate diameter, **F**_**4**_**BMet** in a selected formulation is injected *i.v.* into the tail vein. After appropriate DLI (15 min/24 h), the tumor is irradiated with 42/60 J/cm^2^ light dose of 750 nm laser. Created with Biorender.com (b) Antitumor efficacy of **F**_**4**_**BMet** in GlyGly buffer and formulated in P123 micelles against CT26 tumors in BALB/c mice model at DLI = 15 min (V-PDT) and 24 h (C-PDT), and with different light doses, presented as Kaplan–Meier survival plot. (c) Antitumor efficacy of F_2_BMet against CT26 tumors in BALB/c mice model at DLI = 15 min (V-PDT)–with light dose 60 J/cm^2^, and 24 h (C-PDT) with light dose of 100 J/cm^2^, and with different light doses, presented as Kaplan–Meier survival plot.

Based on noninvasive fluorescence measurement data and real-time in vivo imaging, we explored the antitumor efficiency of V-PDT (DLI = 15 min) as well as the cellular-targeting approach (C-PDT, DLI = 24 h) with **F**_**4**_**BMet** formulated in GlyGly buffer and Pluronic P123 at 1.5 mg/kg BW in the treatment of CT26 tumor-bearing BALB/c mice (Figure 10a). In both cases, photosensitizer was administered via *i.v.* injection in the tail vein and the tumors were exposed to 42 and 60 J/cm^2^ of 750 nm laser 15 min or 24 h post-injection. For comparison, the same in vivo model was treated with redaporfin (F_2_BMet) in different protocols. Redaporfin at dose 1.5 mg/kg BW was administrated via *i.v.* injection into the tail vein and the tumors were exposed to 60 J/cm^2^ after DLI = 15 min, or 100 J/cm^2^ after DLI = 24 h. The therapeutic efficacy was analyzed in terms of mice post-treatment survival time, and the results obtained for the various treatments are presented as Kaplan–Meier plots in Figure 10.

All **F**_**4**_**BMet**-PDT protocols result in delaying tumor progression compared to that of untreated control animals. However, C-PDT was more effective than V-PDT leading to 30% long-term cures for **F**_**4**_**BMet/P123** and ca. 20% for **F**_**4**_**BMet/GlyGly**, respectively. In the case of V-PDT at a lower light dose (42 J/cm^2^) only slight inhibition of tumor growth after treatment with prolonged survival time up to 20 days post-PDT was observed with no cases of cures. Similar effects were observed also after a higher light dose (60 J/cm^2^), increasing the survival time to 25 days and delaying tumor growth. Moreover, V-PDT for each PS formulation did not result in acute inflammatory reactions characteristic of vascular effect, which can be related to relatively low accumulation in tumor tissue immediately after PS injection (DLI = 15 min). Low effectiveness in vascular therapy may also be attributed to the low effectiveness of binding with albumin in the blood.^62^ These results suggest that **F**_**4**_**BMet** is not suitable for the V-PDT approach. In comparison, **F**_**2**_**BMet-PDT** after irradiation with a light dose of 60 J/cm^2^, resulted in the complete eradication of all treated tumors and ultimately 50% long-term cures (Figure 10c).

It is widely recognized that vascular versus cellular targeting is highly dependent upon the biodistribution and pharmacokinetics of photosensitizers.^63^**F**_**4**_**BMet-PDT** at longer DLI can induce more selective tumor damage due to photosensitizer accumulation into the tumor cellular compartment. As shown in Figure 10b, C-PDT with **F**_**4**_**BMet/P123** and 42 J/cm^2^ light dose offers a survival rate of 30%. Redaporfin (**F**_**2**_**BMet**) at DLI = 24 h in a similar model with a higher light dose failed to cure any mice.^7^ When we increased the light dose of C-PDT with redaporfin, we found a slight increase in survival time compared to non-treated mice, but no cures (Figure 10c). Other attempts with redaporfin-C-PDT using DLI between 24 and 72 h and different drug and light doses yielded low cure rates.^7,39,40^ In contrast, **F**_**4**_**BMet** leads to long-term cures after irradiation with relatively low drug and light doses.

Apparently, PDT efficacy is not consistent with fluorescence imaging, which appears to show more **F**_**4**_**BMet**/GlyGly present in tumors than **F**_**4**_**BMet/P123**. The difference between fluorescence imaging and PDT may be related to where **F**_**4**_**BMet** is actually in the tumor tissue. Figure 3 shows that exposure of tumor cells to the free form of **F**_**4**_**BMet** (which is the form it will be present in the tumor milieu after the **F**_**4**_**BMet/GlyGly** administration) or to **F**_**4**_**BMet** encapsulated in micelles does not significantly affect uptake. Initial accumulation of **F**_**4**_**BMet** in the liver could lead to an increased level of elimination of the photosensitizer in the GlyGly formulation. Figure 5 shows that formulations with P123 have enhanced phototoxicity. It is likely that some of the **F**_**4**_**BMet/GlyGly** tends to aggregate when it arrives at the tumor milieu and finds a lower pH environment. This may hinder further diffusion inside the tumor, and the inner tumor cells may not be properly exposed to **F**_**4**_**BMet**. Fluorescence imaging does not discriminate the tumor infiltration of **F**_**4**_**BMet** but this is essential for the efficacy of C-PDT. Fluorescence imaging confirms the presence of **F**_**4**_**BMet** within the tumor, but it does not provide information on whether the compound has penetrated tumor cells. Encapsulation of **F**_**4**_**BMet** not only enhances the production of ROS but also assists the photosensitizer in penetrating deeper into the structure of the tumor. The results obtained in in vivo experiments underline all of the results obtained in in vitro research, presenting **F**_**4**_**BMet** as an effective photosensitizer for targeted PDT and as a selective agent for fluorescence imaging.

## Summary and Conclusions

We explored a new family of fluorinated sulfonamide porphyrin derivatives characterized by the presence of Ph-NHSO_2_R or Ph-NRSO_2_R substituents in the *meso-* positions of the tetrapyrrole ring and fluorination of all other positions of the phenyl rings. We found that the porphyrins with Ph-NRSO_2_R substituents are insufficiently photostable for PDT, but those with Ph-NHSO_2_R substituents have appropriate photostability and singlet oxygen quantum yields. Additionally, we identified a bacteriochlorin, **F**_**4**_**BMet**, with a high fluorescence quantum yield as a promising agent for fluorescence imaging. The porphyrins and the bacteriochlorin-bearing Ph-NHSO_2_CH_3_ groups in all of the *meso-* positions showed low toxicity in the dark. Incorporation of these porphyrin derivatives into Pluronic P123 triblock copolymer micelles increased their phototoxicities. The bacteriochlorin **F**_**4**_**BMet** is especially phototoxic toward a variety of cancer cells, as it generates more ROS, especially hydroxyl radicals, at lower concentration, compared to porphyrins. Encapsulation of **F**_**4**_**BMet** in Pluronic P123 micelles enhanced the generation of hydroxyl radicals. **F**_**4**_**BMet** was selected for in vitro tests with organoids and then for in vivo studies. Moreover, the p*K*_a_ of **F**_**4**_**BMet** is close to 7, which allows for solubilization in a glycylglycine buffer.

The biological effects of **F**_**4**_**BMet** were examined on hiPSC-derived CRC organoids, which served as models of human colon tumors in two formulations: GlyGly buffer and P123 micelles. We found low cytotoxicity in the dark and high phototoxicity, especially for **F**_**4**_**BMet/P123**. It seems that **F**_**4**_**BMet/GlyGly** tends to accumulate in the periphery of the organoid structure.

The biodistribution of **F**_**4**_**BMet** was investigated using noninvasive fluorescence measurements. **F**_**4**_**BMet** exhibited accumulation in the tumor 3 h after injection, and in the case of the GlyGly formulation, this photosensitizer remained detectable in the tumor area for up to 72 h postinjection. These results suggest that **F**_**4**_**BMet** can be used as an agent for fluorescence imaging or for image-guided PDT. **F**_**4**_**BMet** enabled the development of an efficient C-PDT protocol for CT26 tumor-bearing BALB/c mice with a DLI of 24 h. The **F**_**4**_**BMet** dissolved in an aqueous pH-controlled solution (GlyGly buffer) combined with the light dose of 42 J/cm^2^ at DLI = 24 h led to ca. 20% of long-term cures. The higher in vivo efficacy was observed when **F**_**4**_**BMet** was encapsulated in P123 micelles (ca. 30% long-term cures) with no observable adverse effects related to the photosensitizer formulation. This is consistent with the photodynamic effects of **F**_**4**_**BMet/GlyGly** and **F**_**4**_**BMet/P123** on CRC organoids.

In conclusion, among the series of obtained and characterized photosensitizers, the nanoformulated sulfonamide perfluorinated bacteriochlorin offers the greatest theranostic potential. It proved to be a selective NIR fluorescence imaging agent for the detection of tumors and allowed us to expand the use of bacteriochlorins to C-PDT. Excelling in fluorescence imaging and in C-PDT, **F**_**4**_**BMet** closely approaches the properties of the ideal photosensitizer for image-guided targeted PDT.

## Experimental Section

### Theoretical Calculations

In all electronic structure calculations, DFT methods as implemented in ORCA^64^ were used due to their lower computational cost compared to wave function-based methods. All geometries were optimized using the PBE0 hybrid functional^65^ with the triple-ζ Pople 6-311G* basis set.^66^ Harmonic frequency analyses were performed for each structure to ensure that a minimum energy configuration was considered. Solvent effects were simulated using the Conductor-like Continuum Polarization Model (C-PCM),^67^ with the dielectric constant and refractive index as the data default in ORCA for water (Figures S2 and S3, Tables S1–S3, Supporting Information).

### Synthesis

#### 5,10,15,20-Tetrakis-[2′,3′,5′,6′-tetrafluoro-4′-methanesulfamoyl)phenyl] Bacteriochlorin F_4_BMet

The bacteriochlorin was prepared using the solvent-free methodology.^36^ A mixture of F_4_PMet (20 mg, 0.016 mmol) and *p*-toluenosulfonylhydrazide (117 mg, 0.63 mmol) was grounded in a Schlenk tube and then evacuated with a vacuum pump. It should be noted that there is a danger of spontaneous deflagration of *p*-toluenosulfonylhydrazide under an atmosphere of nitrogen above 127 °C.^68^ Next, the reactor was heated at 140 °C for 40 min and then brought back to room temperature. After chromatography with silica gel (dichloromethane/ethyl acetate 3:1), **F**_**4**_**BMet** was obtained as a green power (14 mg, 70%). ^1^H NMR (400 MHz, (CD_3_)_2_CO): δ = 8.46 (s, 4H), 4.27 (s, 8H), 3.38 (s, 12H), −1.24 ppm (s, 2H), (Figure S11, Supporting Information); ^19^F NMR (376.5 MHz, (CD_3_)_2_CO, TFA as external reference) δ −141.19 (dd, *J* = 23.7, 10.4 Hz), −146.70 (dd, *J* = 23.6, 10.4 Hz), (Figure S12, Supporting Information). HRMS (ESI-TOF): *m*/*z* [M + H] ^+^calculated for C_48_H_31_F_16_N_8_O_8_S_4_: 1279.0870, found: 1279.0887 (Figure S13, Supporting Information).

### Spectroscopy

Electronic absorption spectra were recorded on a Hitachi U-2001 or Shimadzu 2100 spectrophotometer. Fluorescence spectra were recorded in a Horiba-Jovin-Yvon Spex Fluorog 3–2.2 spectrophotometer in a 1 cm quartz cuvette and corrected for the detector wavelength dependence. Porphyrins fluorescence quantum yields were determined for samples diluted 10-fold from an initial solution with 0.2 of absorbance in the Soret band. The fluorescence standard was *meso-*tetraphenylporphyrin (TPP) Φ_F_ = 0.10 ± 0.01.^51^ The fluorescence quantum yield of bacteriochlorin (**F**_**4**_**BMet**) was determined using the portable spectrometer Avantes model coupled to a CW laser (λ = 748 nm) and a cuvette holder through an optical fiber. The emission was collected at a right angle from excitation; the fluorescence standard was HITCI (Φ_F_ = 0.283) molecule.^67^ Transient absorptions were recorded in an Applied Photophysics LKS.60 laser flash photolysis spectrometer with a Hewlett-Packard Infinium Oscilloscope and a Spectra-Physics Quanta-Ray GCR-130 Nd:YAG laser as the excitation source. Samples were irradiated with the third harmonic of the laser at 355 nm (*E*_max_ = 30 mJ per pulse and full width at half-maximum, fwhm = 6 ns). At least five kinetic runs were registered under each set of conditions and their average was recorded. The measurements were done at 20 °C. All solutions were freshly prepared before the measurements with an absorbance of about 0.2 at 355 nm. For the experiments in the absence of oxygen, the solutions were purged with nitrogen for 20 min until no changes in the decay rate were observed. The lifetime fittings were analyzed using monoexponential decays. The same apparatus and excitation wavelength were used in the determination of singlet oxygen quantum yields using phenalenone as a reference. Singlet oxygen phosphorescence was collected at 1270 nm through a Hamamatsu R5509–42 photomultiplier, cooled to 193 K in a liquid nitrogen chamber using a filter (Newport model 10LWF-1000-B) to block the porphyrins fluorescence. These measurements were made in ethanol.

### Photodecomposition Quantum Yields

Photodecomposition experiments were made by dissolving the photosensitizers in methanol:water (3:2, V, V) solution. The volume of 3 mL of solution was placed in a cuvette and irradiated, ensuring that all the light hits the solution. The porphyrins were irradiated at 423 nm and output power of 10 mW using a Nd:YAG pulsed laser (ca. 6 ns) EKSPLA model NL301G coupled an OPO EKSPLA model PG/122/SH. The **F**_**4**_**BMet** was irradiated using a CW laser (Omicron) with emission at 748 nm and output power of 50 mW/cm^2^. The volume lost by evaporation during the irradiation was compensated for by weighting the cuvettes and adding methanol to the sample before each measurement.

Photodecomposition quantum yield (Φ_PD_) is defined as the ratio between the rate of disappearance of photosensitizer molecules *V*_d_ and the rate of absorption of photons *V*_p_:
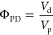
Where:
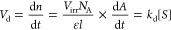

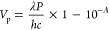
*k*_d_ is the decomposition rate constant and [*S*] the concentration of the photosensitizer. *P* is the power of the monochromatic incident light absorbed in volume (*V*_irr_) by a solution of absorbance *A*.

### Determination of Partition Coefficient (Log *P*_OW_)

The so-called shaking method was used to determine log *P*. A small amount of the compound was dissolved in 5 mL of n-octanol saturated with PBS buffer, and then the resulting solution was sonified for 10 min. Then 5 mL of PBS was saturated with n-octanal and the resulting system was also sonified. The mixture was shaken and stirred using a vortex shaker for 15 min. From both phases 0.01 mL was taken and diluted with 1.99 mL of DMSO. The solution thus obtained was also sonified, and then the fluorescence spectra were measured. In order to determine the PS concentration, a calibration of the method used was performed. A calibration curve was drawn up based on the fluorescence intensity obtained for a series of solutions of the photosensitizers in DMSO containing, respectively, 0.5% PBS buffer or octanol in the concentration range *c* = 0.1 μM – 1 nM (Figure S4, Supporting Information).

### Detection of Reactive Oxygen Species in Solution

SOSG, APF, and HPF probes were acquired from Invitrogen and SigmaAldrich. Photosensitizer solutions were diluted to a final concentration of 5 μM per well in PBS with DMSO content <0.5%. Next, a given fluorescent probe was added to each well at a final concentration of 10 μM. The solutions were irradiated with 635 ± 20 nm (for porphyrins) and 735 ± 20 nm (for bacteriochlorin) laser diode light (Instutut Fotonowy, Poland) for various time intervals. In all experiments, the light dosimetry was performed and controlled using Ophir NOVA II radiometer (Laser Measurements Group). The microplate reader (Tecan Infinite M200 Reader) was used for the acquisition of fluorescence signals immediately before and after illumination. When APF and HPF were employed, excitation was at 490 nm and the fluorescence emission was detected at 515 nm, whereas the corresponding values for SOSG were 505 and 525 nm, respectively.

Hydroxyl radical also forms adducts with DMPO, which were identified by electron paramagnetic resonance (EPR). EPR measurements were performed at room temperature by using a Bruker ESP 300 spectrometer (IBM Instruments Inc.). The EPR spectra were recorded under in situ irradiation with a Hamamatsu diode laser (ref LA0873 S/N M070301) delivering 134 mW at 748 nm and controlled by a ThorLabs 500 mA ACC/APC Laser Diode Controller.

### Cells Culture

The in vitro studies employed human lung adenocarcinoma (A549), murine colon carcinoma (CT26), and murine skin melanoma (B16–F10) cell lines. A549 and CT26 cells were grown in DMEM ented with 4.5 g/L glucose (BioTech) and B16–F10 cells in RPMI 1640 (Sigma-Aldrich) with the addition of 10% fetal bovine serum (BioTech, Poland) and 1% of antibiotics (penicillin/streptomycin). Before the experiments, the cells were removed by trypsinization, washed with PBS, and maintained in a humidified atmosphere at 37 °C and 5% CO_2_.

### Cellular Uptake Studies

The cells for uptake of the photosensitizers were seeded on a 96-plate microplate (10^4^ per well). After 24 h, the cells were incubated with 20 μM solution of porphyrins or 5 μM solution of bacteriochlorin for various time intervals (from 2 to 24 h). The solutions of photosensitizers were prepared by diluting the photosensitizer stock solution in DMSO. The highest concentration of DMSO in the medium did not exceed 0.5%. The formulations with Pluronic P123 were prepared by the thin-film hydration method as described elsewhere.^46^ The particle sizes of photosensitizers encapsulated in Pluronic P123 micelles were measured by DLS using a ZetaSizer (Malvern Instruments). After incubation with each photosensitizer, the cells were washed two times with warmed PBS with Ca^2+^ and Mg^2+^ and solubilized in 30 μL of Triton X-100 and 70 μL of DMSO/ethanol solution (1:3). The retention of cell-associated porphyrin was detected by fluorescence measurement with a microplate reader (Tecan Infinite M200 Reader).

### Fluorescence Confocal Images

Confocal images were taken with a LSM780 Zeiss Confocal Microscope. For **F**_**4**_**BMet,** a 405 nm wavelength laser was used, and the fluorescence was observed over the 750 nm wavelength. For DAPI, a 355 nm laser was used, and the emission was observed at the 450–550 nm wavelength range. 63*x*/1.4 oil lens was used.

### Viability Test and Cytotoxicity in the Dark

The AlamarBlue (resazurin) assay was used to measure cell survival and assess cytotoxicity in the dark and phototoxicity. Photosensitizer solutions were prepared with concentrations up to 100 μM in the growth medium and, after cell attachment, added to the cell cultures. Treated cultures were incubated for 18–20 h in the dark (optimal uptake time was determined experimentally). Next, the photosensitizer solution of each well was removed, cells were washed in PBS with Ca^2+^ and Mg^2+^ and fresh culture medium supplemented with FBS, and antibiotics were added to each well. Then, the cells were returned to the incubator for 24 h. After this time, a viability test was performed. Resazurin stock solution was prepared by dissolving 0.05 g Resazurin sodium salt into 10 mL 1× PBS. Briefly, 10 μL of Resazurin solution were added to each well and the microplates were further incubated for 3 h. Resazurin quantification was performed using an automatic microplate reader (Tecan Infinite M200 Reader) by fluorescence measurements with a 605 nm test wavelength.

### Photodynamic Effect

Based on cytotoxicity results, nontoxic concentrations (20 μM for **F**_**4**_**TPP-MS** and **F**_**4**_**PMet**, 5 μM for **F**_**4**_**BMet**) were selected for the study of PDT efficacy in vitro. Cells were incubated in the dark with a photosensitizer solution (0.5% DMSO or P123 micelles in PBS) in a culture medium for 18–20 h. After this incubation the cells were washed twice with PBS with Ca^2+^ and Mg^2+^, then the 100 μL of PBS with Ca^2+^ and Mg^2+^ was added to each well, and cells were irradiated with the 635 nm ± 20 nm red light for porphyrins or 735 nm ± 20 nm NIR light for bacteriochlorin, respectively. Next, the cells were washed with PBS with Ca^2+^ and Mg^2+^, fresh growth medium was added, and the plates were returned to the incubator for 24 h. Cell viability was determined with the AlamarBlue assay 24 h after the irradiation using the procedure described above. Images from the cell morphology before and after photodynamic treatment were captured using an Olympus IX51 microscope equipped with TH4–20 (Olympus), processed using the cellSens Imaging software (Olympus), and elaborated with the ImageJ software and using a LSM780 Zeiss Confocal Microscope equipped with 63*x*/1.4 oil lens. For Hoechst33258 a 355 nm laser was used for excitation, and the emission was observed at the 450–550 nm wavelength range, and PI was visualized in the 600–650 range after excitation with 561 nm wavelength.

### Hydroxyl Radical Generation In Vitro

A nontoxic concentration of **F**_**4**_**BMet** was selected −5 μM. Solution of **F**_**4**_**BMet/P123** was prepared in PBS. CT26 cells were incubated in the dark with photosensitizer (0.5% DMSO or with micelles) in a culture medium for 18–20 h. Two hours before the end of incubation APF (Invitrogen) was added with a final concentration of 10 μM. Next, the cells were washed with PBS with Ca^2+^ and Mg^2+^, and fresh PBS was added to the plates. The plate was irradiated with 735 ± 20 nm NIR light. The fluorescence of fluorescein was assessed using an automatic microplate reader (Tecan Infinite M200 Reader, excitation: 495 nm; emission: 515 nm).

### CRC Organoids

hiPSCs were cultured on Geltrex (Thermo Fisher) precoated 6-well plates in mTeSR1 growth medium (STEMCELL Technologies). Cells were grown in an incubator at 37 °C with 5% CO_2_. The iPSC differentiation to colonic organoids as a model of CRC was performed using the experimental conditions and protocols described in Crespo et al.^68^ This method is based on the modulation of signaling pathways for the progressive generation of (1) DE with CHIR99021 and activin A, (2) hindgut endoderm (HE) using CHIR99021 and FGF4, and subsequently, colonic organoids (CO) through supplementation with CHIR99021 + LDN19318 + EGF and B27. The time needed for the complete differentiation process reached ca. 40 days. Throughout the ensuing 6 weeks, individual colonic stem cells produced colonic organoids (COs), which were passaged at a 1:4 density every 10 days. Three days before the assay, COs were switched to a colonic medium without CHIR.

### Uptake

Organoids were seeded on a 96-plate microplate. After 3 days, they were incubated with photosensitizers (2 mg/kg BW) for time intervals from 2 up to 24 h. After incubation, organoids were washed twice with PBS and lysed with RIPA buffer and solubilized in 30 μL of Triton X-100 and 70 μL of DMSO/ethanol solution (1:3). The retention of the cell-associated photosensitizer was detected by fluorescence with the microplate reader (Tecan Infinite M200 Reader).

### Cytotoxicity in the Dark

The CellTox Cytotoxicity Assay (Promega) was used to quantify cell survival and photosensitizer-mediated cytotoxicity. Organoids were placed in Matrigel on a 96-plate glassed-bottom microplate. After 3 days, they were incubated with photosensitizers in a growth medium at concentrations from 0 to 5 mg/kg BW in the dark. Next, the solution of each well was removed, cells were washed in PBS, and fresh culture medium was added to each well, and cells were returned to the incubator for 24 h. The CellTox Green Cytotoxicity Assay was performed, and quantification was made using an automatic microplate reader (Tecan Infinite M200 Reader) by fluorescence measurements.

### Photodynamic Effect

On the cytotoxicity results, a nontoxic concentration of photosensitizers (2 mg/kg BW) was selected. Organoids were incubated for 24 h in the dark with a photosensitizer solution in a culture medium. After this incubation time, the organoids were washed with PBS and irradiated with a 735 ± 20 nm LED irradiation system for various time intervals. Next, the organoids were washed with fresh medium, and the plates were returned to the incubator for 24 h. Organoids viability was determined by the CellTox Green Cytotoxicity Assay in independent experiments performed 24 h postirradiation.

### Animal Model

All experiments were carried out with approval nos. 42/2014, 190/2018 and 242/2022 of the first Ethics Committee for Research on Animals, Krakow, Poland. BALB/c mice (8–9-weeks-old males, AnimaLab) were used to perform the in vivo evaluation. The animals were humanely treated and supplied with food and water *ad libitum*. The animals were housed and maintained in individually ventilated cages under a 50–60% humidity, 12/12 h light/dark cycle and at 22 ± 2 °C, in SPF conditions at the Faculty of Biochemistry, Biophysics and Biotechnology, Jagiellonian University in Krakow, Poland.

### Noninvasive Fluorescence Detection

The CT26 cells (0.5 × 10^6^ in PBS suspension) were implanted subcutaneously into the right thigh of BALB/c mice. The *i.v*. administration of the drug formulation was done when the tumor attained a diameter of 5–6 mm in each animal, which usually took about 7 days after inoculation. Each formulation (**F**_**4**_**BMet** dissolved in GlyGly buffer as well as Pluronic P123 micelles), corresponding to 1.5 mg/kg BW of photosensitizer was injected into the tail vein of each animal, and fluorescence measurements were performed at selected time intervals (0–96 h) after PS *i.v.* injection. Fluorescence spectra of **F**_**4**_**BMet** were collected from the tumor and skin area using an LS55 spectrofluorimeter (PerkinElmer) equipped with fiber optics. The spectra were registered using λ_exc_ = 510 nm and λ_em_ = 720–780 nm.

### Real-Time Whole-Body Imaging

CT26 tumor cells (0.5 × 1 × 10^5^ in PBS suspension) were implanted on the right thigh of BALB/c mice. Tumor growth monitoring began 1 week after cell implantation and continued every day for 10 days. When the tumors reach ca. 0.5 cm in diameter, the mice were injected *i.v.* with **F**_**4**_**BMet/P123** or **F**_**4**_**BMet/GlyGly** with dose of 1.5 mg/kg body weight, and whole-body imaging using the Newton 7.0 Imaging System (Vilber) was performed immediately following and up to 72 h after the injection under inhalation anesthesia with 3–4% Isoflurane (Aerrane, Baxter, Poland) throughout the experiment. Fluorescence images of BALB/c mice were collected using λ_exc_ = 690 nm and λ_em_ = 740–780 nm to illustrate the specific signal for investigated bacteriochlorin.

### Photodynamic Therapy

The antitumor efficacy of **F**_**4**_**BMet** in GlyGly buffer and P123 micelles, and **F**_**2**_**BMet** was evaluated in BALB/c mice bearing CT26 tumors implanted subcutaneously in the right leg. When the tumor volume reached about 4–5 cm in diameter, mice were randomly assigned to experimental groups (*n* = 6–9). **F**_**4**_**BMet/F**_**2**_**BMet** was administered via the tail vein injection with a dosage of 1.5 mg kg^–1^ BW. The tumor irradiation was performed at DLI = 15 min or DLI = 24 h using a laser (Omicron laser model LDM750.300.CWA.L.M equipped with optical fiber model FD/Medlight, Ecublens, Switzerland) at 748 nm, radiant exposures 42, 60 or 100 J/cm^2^ and a laser power of 50 mW. The illuminated area with a diameter of 1.3 cm was kept constant during irradiation. Survival curves were estimated employing the Kaplan–Meier analysis.

### Statistical Analysis

The STATISTICA software for biostatistics (StatSoft Inc.) was used for statistical analysis of the data. The data are expressed as means ± standard deviation or standard error of the mean (SEM) of at least three independent experiments. The difference between groups was evaluated with the student’s *t* test and considered significant for *p* < 0.05.

## Supporting Information Available

Electronic absorption, emission spectra of porphyrin derivatives, theoretical studies of HOMO, LUMO orbitals of fluorinated and nonfluorinated bacteriochlorin with calculated geometries and orbital energies, examples of fitting curves for determination of experimental values of Log *P* with calculated Partition Coefficient values for three selected compounds, transient absorption spectra of **F**_**4**_**BMet** with triplet state decays, DLS data of acquired micellar formulation, confocal fluorescence imaging of CT26 with accumulated **F**_**4**_**BMet**, confocal imaging of various cancer cell lines showing changes in morphology, confocal fluorescence imaging of live/dead CT26 cells before/after PDT, and bacteriochlorin characterization through NMR and mass spectroscopy (PDF)

## Abbreviations

4T1: murine breast cell line
A549: human lung carcinoma cell line
APF: aminophenyl fluorescein
B16–F10: murine melanoma cell line
BW: body weight
COs: colonic organoids
C-PDT: cellular-targeted photodynamic therapy
CT26: murine colon tumor cell line
DLI: drug-to-light interval
DLS: dynamic light scattering
DMSO: dimethyl sulfoxide
eM6: human fibroblast cell line
EPR: enhanced permeability and retention
FBS: fetal bovine serum
FDA: U.S. Food and Drug Administration
GlyGly: glycylglycine buffer
hiPSC: human induced pluripotent stem cells
HITCI: 1,1′,3,3,3′,3′-Hexamethylindotricarbocyanine iodide
HOMO: highest occupied molecular orbital
HPF: hydroxyphenyl fluorescein
*i.v.*: intravenous
IC_50_: half maximal inhibitory concentration
IL: interleukin
mRNA: mRNA
NIR: near-infrared
NMR: nuclear magnetic resonance
PBS: phosphate buffered saline
PDI: photodynamic inactivation of microorganisms
p*K*_a_: acid dissociation constant
P_OW_: n-octanol–water partition coefficient
PS: photosensitizer
RT: real-time
SOSG: singlet oxygen sensor green
TFA: terephthalic acid
TME: tumor microenvironment
TNFα: tumor necrosis factor α
VEGF: vascular endothelial growth factor
V-PDT: vascular-targeted photodynamic therapy
ε: molar absorption coefficient
λ_Ex_: excitation wavelength
λ_Em_: emission wavelength
τ_T_: triplet state lifetime
Φ_F_: fluorescence quantum yield
Φ_Δ_: singlet oxygen quantum yield
Φ_PD_: photodecomposition quantum yield
