# Supplementary material for: Better in the Near Infrared: Sulfonamide Perfluorinated-Phenyl Photosensitizers for Improved Simultaneous Targeted Photodynamic Therapy and Real-Time Fluorescence Imaging
Source: ACS Appl Mater Interfaces. 2024 Sep 14;16(38):50389–406. doi: 10.1021/acsami.4c11171 (PMC11440460; doi:10.1021/acsami.4c11171)
Supplement: Supplementary file 1 — am4c11171_si_001.pdf [file am4c11171_si_001.pdf]

Supporting Information:

Better in the Near Infrared: Sulfonamide Perfluorinated-Phenyl Photosensitizers for Improved Simultaneous Targeted Photodynamic Therapy and Real-Time Fluorescence Imaging

Marta Warszyńska,[a,b] Barbara Pucelik,[c] Carolina S. Vinagreiro,[d] Paweł Repetowski, [a,b] Agata Barzowska,[c] Dominik Barczyk,[a,b] Fábio A. Schaberle,[d] Amilcar Duque-Prata,[d] Luis G. Arnaut,[d] Mariette M. Pereira [d] and Janusz M. Dąbrowski [a]

[a]Faculty of Chemistry, Jagiellonian University, 30-387 Kraków, Poland

[b]Doctoral School of Exact and Natural Sciences, Jagiellonian University, 30-348 Kraków, Poland

[c]Łukasiewicz Research Network – Kraków Institute of Technology, ul. Zakopiańska 73, 30-418, Kraków, Poland

[d]CQC-IMS, Department of Chemistry, University of Coimbra, 3004-535 Coimbra, Portugal

*Correspondence should be sent to Janusz M. Dąbrowski, jdabrows@chemia.uj.edu.pl, +48126632293, +48126340515 (fax)

Table of Contents

Figure S1 Electronic absorption and fluorescence spectra…………………..……..………..…3

Figure S2 HOMO and LUMO orbitals of H_4Bmet.………………...….…………..…………3

Figure S3 HOMO and LUMO orbitals of F_4BMet ……….………..……………….……….…...4

Table S1 Geometry of H_4BMet ……..………..……………………..………..……...………..5

Table S2 Geometry of F_4BMet ……..………..…………………………..……..………..…..…..7

Table S3 Orbital energies ……..……..………………….……………..……..……….………9

Figure S4 Fitting curves of LogP ……………………………………………..……..……9

Table S4. LogP values were determined for F_4TPP-MS, F_4PMet and F_4Bmet………………10

Figure S5 Transient absorption spectra of F_4BMet.……………………………………..…10

Figure S6 Triplet state decays of the F_4BMet …..……………….………………..……...……..10

Figure S7 Dynamic Light Scattering ……………………………..…..………………..11

Figure S8 Confocal fluorescence imaging ………………………...…………...…………12

Figure S9 Photodynamic effect against A549, B16F10 and CT26 cells…………………..…13

Figure S10 The red pixels quantification after photodynamic effect on organoids….………14

Figure S11 ^{1}H NMR spectra of F_4BMet ....………………..……..…………..…….……..15

Figure S12 ^{19}F NMR spectra of F_4BMet …..………………………………...……………16

Figure S13 ESI-FIA-TOF Mass Spectra for F_4BMet……………………………………17

Spectroscopic characterization of synthesized photosensitizer

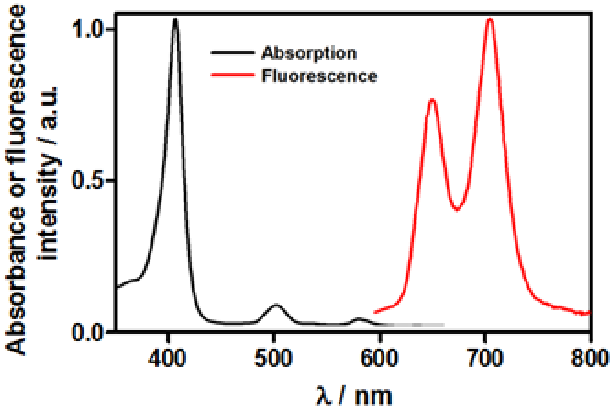

Figure S1. Electronic absorption and emission spectra of selected porphyrin derivative (F_4PMet) recorded in toluene. Emission spectrum obtained after excitation with 488 nm wavelength.

Theoretical studies of studied PS

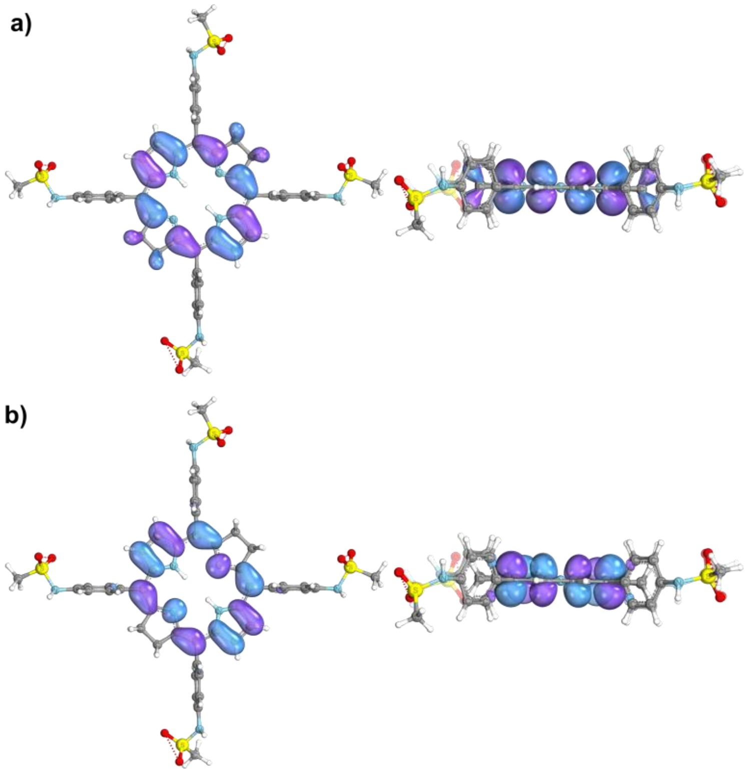

Figure S2. HOMO (a) and LUMO (b) orbitals of **H_4BMet** computed at PBE0/6-311G* level of theory, with C-PCM considering water as the solvent.

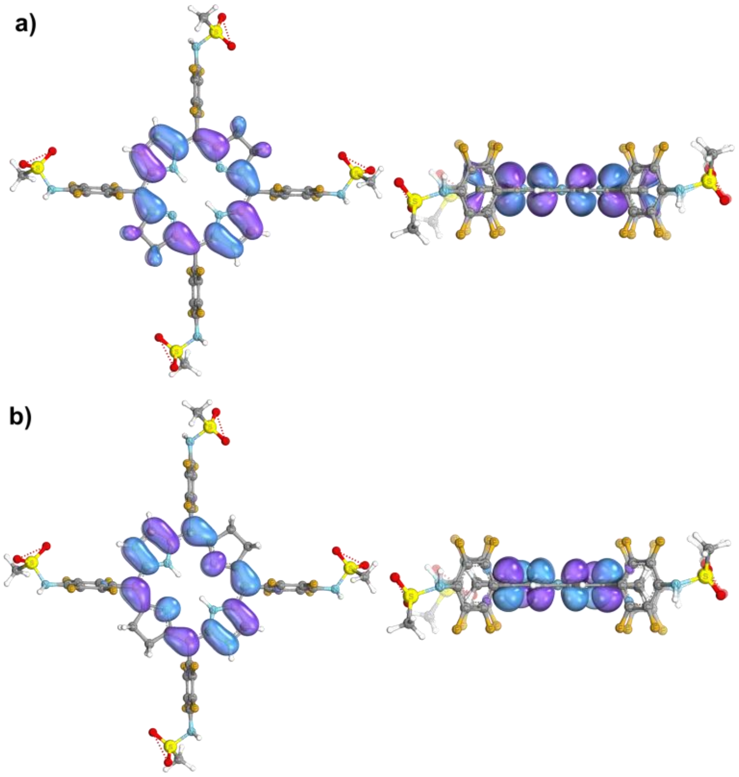

Figure S3. HOMO (a) and LUMO (b) orbitals of **F_4BMet** computed at PBE0/6-311G* level of theory, with C-PCM considering water as the solvent.

Table S1. Geometry of **H_4BMet** computed at PBE0/6-311G* level of theory, with C-PCM considering water as the solvent.

C	-3.358678277	1.684895734	0.736537008
C	-2.416833236	2.656143035	0.965114437
C	-2.680450906	0.483408144	0.391793617
C	-1.13100455	2.079835692	0.772927211
N	-1.339753268	0.769368342	0.426828628
H	-0.608127513	0.104627195	0.230241245
C	-3.276834917	-0.749941463	0.08272751
C	-2.624268591	-1.932925209	-0.261850133
C	-3.391315815	-3.19924605	-0.579414659
C	-2.286083159	-4.181686376	-0.94245471
H	-4.102963282	-3.035863155	-1.393191742
H	-3.977518478	-3.52647478	0.28386833
C	-1.028354106	-3.365643745	-0.731649008
H	-2.355199035	-4.529299978	-1.977116939
H	-2.285858809	-5.076737306	-0.314652049
N	-1.290508235	-2.097993691	-0.358302844
C	0.239119075	-3.918439449	-0.911575094
C	1.473459589	-3.269842515	-0.743861143
C	2.759999685	-3.848748988	-0.924652115
C	3.702153002	-2.886748139	-0.661854695
C	3.023497011	-1.685625505	-0.316392961
N	1.682314331	-1.96441659	-0.37913062
H	0.95038555	-1.299630154	-0.183877719
C	3.620575545	-0.460656487	0.022126118
C	2.967964632	0.72594444	0.353335916
C	3.736038324	1.985056423	0.695186293
C	2.628125874	3.001034352	0.938567224
H	4.406617528	2.271323554	-0.119507572
H	4.366518467	1.829539888	1.575228822
C	1.370829654	2.179622544	0.746380956
H	2.658648364	3.835908464	0.232641502
H	2.663883104	3.440157727	1.939120244
N	1.633532824	0.902294183	0.407879847
C	0.103246165	2.734073408	0.916673658
H	2.937161454	-4.872913747	-1.218060404
H	4.775829151	-2.995892684	-0.704224982
H	-4.431839703	1.788743566	0.800149752
H	-2.593998915	3.684078192	1.245259867
C	-4.770422837	-0.781702498	0.131789443
C	5.115344435	-0.441740511	0.020221203
C	-5.435625347	-1.243416263	1.268066196
C	-6.823547099	-1.272767597	1.319158233
C	-7.571191425	-0.859981258	0.218048656
C	-6.920097824	-0.408313707	-0.928162013
C	-5.532784571	-0.359417149	-0.959740894
H	-4.862175058	-1.57461525	2.128688712
H	-7.330055271	-1.620736256	2.214740778
H	-7.501233737	-0.100930612	-1.789591981
H	-5.032138564	-0.004423872	-1.855581911
C	5.825682364	-0.191306523	-1.154310783
C	5.833336943	-0.679691275	1.194227939
C	7.22171391	-0.675358401	1.200282932
C	7.917849359	-0.403128374	0.024027549
C	7.214716271	-0.159364708	-1.154161751
H	5.297315654	-0.890343358	2.114887522
H	7.756625521	0.050845038	-2.071764777
H	5.286859821	-0.008780654	-2.079174499
H	7.768687418	-0.886471899	2.111396259
C	0.035149207	4.177010177	1.301813579
C	-0.031438299	4.554870876	2.644768868
C	-0.105063142	5.891825703	3.011369998
C	-0.084831462	6.881258646	2.030155452
C	-0.014288067	6.519114578	0.686339682
C	0.032853928	5.17710485	0.32913231
H	0.077239007	4.904959529	-0.72117371
H	-0.002691718	7.289100546	-0.079644493
H	-0.043300076	3.789590896	3.415099877
H	-0.181158632	6.172473808	4.05515823

C	0.308326436	-5.355980764	-1.314537208
C	0.200927147	-5.732898639	-2.654242819
C	0.486576812	-6.358611794	-0.360770353
C	0.276637897	-7.065217291	-3.037827231
C	0.435374897	-8.058896874	-2.072534766
C	0.537247647	-7.696394763	-0.729683158
H	0.575034088	-6.092389917	0.688289967
H	0.074338089	-4.969551357	-3.416289606
H	0.221624594	-7.321432082	-4.089898406
H	0.660250907	-8.465856332	0.02704033
N	-8.993433007	-0.959596892	0.23767744
N	-0.20423847	8.253241223	2.399049062
N	9.344452984	-0.434725947	0.015555805
N	0.559500007	-9.422864248	-2.425629617
S	-9.901850963	0.453444241	0.353381011
H	-9.328388575	-1.627099013	0.926041361
S	1.110785331	9.013528544	3.12674523
H	-0.545350746	8.833311768	1.638280464
S	10.16275647	0.843665605	0.749334077
H	9.716688776	-0.569395079	-0.920079331
S	-0.562311044	-10.23844272	-3.359464802
H	0.900389528	-10.02767568	-1.688378816
O	2.032051113	9.544987074	2.124880038
O	1.647539585	8.088804224	4.115346945
O	10.17706692	2.029102707	-0.105136659
O	9.610997915	0.991081669	2.0893943
O	-0.701852801	-11.55352073	-2.746444777
O	-1.741956067	-9.405718583	-3.543281031
O	-10.09494194	0.841873198	1.748587912
O	-9.293383893	1.42743702	-0.541995851
C	0.305854174	10.36658254	3.933936206
H	-0.207500948	10.96861576	3.184742369
H	1.088644596	10.95098027	4.417919722
H	-0.392315287	9.966118186	4.66596608
C	11.7977368	0.17387593	0.832450507
H	12.14042849	-0.048567162	-0.177689337
H	11.77516105	-0.721262277	1.450429193
H	12.42402549	0.944208368	1.282740879
C	0.239635983	-10.44151224	-4.924943396
H	1.161756887	-10.99792708	-4.765152842
H	0.443883545	-9.460629773	-5.351135496
H	-0.445108757	-11.00091152	-5.562972262
C	-11.4502869	-0.093267111	-0.304091174
H	-11.81690136	-0.921329715	0.301836415
H	-11.29882927	-0.396295137	-1.338021284
H	-12.13313338	0.754249049	-0.240140492

Table S2. Geometry of **F_4BMet** computed at PBE0/6-311G* level of theory, with C-PCM considering water as the solvent.

C	-3.3475	1.64649	0.85561
C	-2.4138	2.63482	1.02964
C	-2.6616	0.45109	0.50297
C	-1.1286	2.07236	0.79403
N	-1.3272	0.7546	0.48199
H	-0.5944	0.09856	0.2615
C	-3.2434	-0.795	0.22461
C	-2.5939	-1.97553	-0.13134
C	-3.3531	-3.25505	-0.40195
C	-2.2479	-4.21797	-0.81718
H	-4.1097	-3.1142	-1.17807
H	-3.878	-3.59091	0.49704
C	-0.9996	-3.38029	-0.65396
H	-2.356	-4.55404	-1.85269
H	-2.2109	-5.11564	-0.19469
N	-1.2641	-2.11689	-0.27553
C	0.27103	-3.90223	-0.8869
C	1.50342	-3.24066	-0.77791
C	2.78933	-3.80625	-1.00231
C	3.72614	-2.83024	-0.78113
C	3.0408	-1.63729	-0.42051
N	1.70441	-1.93202	-0.43123
H	0.97166	-1.27513	-0.21301
C	3.62397	-0.3985	-0.11551
C	2.97328	0.78476	0.22798
C	3.73444	2.05405	0.53709
C	2.6182	3.06914	0.74613
H	4.41249	2.32573	-0.27555
H	4.34821	1.92437	1.43374
C	1.37291	2.21673	0.64707
H	2.61493	3.84286	-0.0277
H	2.6813	3.5823	1.70873
N	1.6406	0.93767	0.32874
C	0.1012	2.74367	0.86454
H	2.97301	-4.83	-1.29437
H	4.79918	-2.92823	-0.85914
H	-4.4185	1.73754	0.96398
H	-2.5991	3.66524	1.29628
C	-4.7296	-0.84154	0.3096
C	5.11164	-0.35514	-0.17432
C	-5.3823	-1.32721	1.43524
C	-6.7622	-1.35317	1.52238
C	-7.5584	-0.91781	0.46667
C	-6.9114	-0.44747	-0.6713
C	-5.5308	-0.40041	-0.73673
F	-4.6733	-1.77312	2.47324
F	-7.3455	-1.81118	2.63123
F	-7.629	-0.03562	-1.71689
F	-4.9675	0.06789	-1.85082
C	5.78873	-0.15386	-1.37031
C	5.88886	-0.5078	0.96702
C	7.27063	-0.47433	0.92235
C	7.94184	-0.25453	-0.27609
C	7.16934	-0.08901	-1.42212
F	5.29997	-0.70438	2.14749
F	7.77703	0.12631	-2.59001
F	5.10371	0.00625	-2.50279
F	7.96513	-0.65768	2.04566
C	0.02544	4.19146	1.20685
C	-0.0783	4.62837	2.52242
C	-0.1513	5.97229	2.84264
C	-0.0954	6.9468	1.85115
C	0.02255	6.51689	0.53233
C	0.06493	5.17029	0.22141
F	0.17763	4.81839	-1.06063
F	0.08608	7.42378	-0.44392
F	-0.1244	3.73876	3.51518
F	-0.273	6.33512	4.12013

C	0.34704	-5.332	-1.29886
C	0.23491	-5.71089	-2.63074
C	0.53829	-6.34896	-0.3725
C	0.32507	-7.03304	-3.02623
C	0.50452	-8.04793	-2.09198
C	0.60081	-7.67704	-0.75367
F	0.64688	-6.05533	0.92348
F	0.04968	-4.78219	-3.56971
F	0.24167	-7.33421	-4.32262
F	0.77066	-8.62292	0.17189
N	-8.9546	-1.00025	0.5336
N	-0.2011	8.31015	2.15775
N	9.34021	-0.25294	-0.345
N	0.641	-9.38694	-2.4775
S	-9.9009	0.39975	0.55059
H	-9.3144	-1.68531	1.18746
S	1.0566	9.10327	2.96624
H	-0.5696	8.8883	1.41006
S	10.2432	0.95931	0.41064
H	9.72397	-0.46883	-1.2574
S	-0.6486	-10.2252	-3.17641
H	1.13451	-9.97493	-1.81641
O	1.36824	10.29264	2.18629
O	2.09542	8.12209	3.23831
O	11.1752	1.46149	-0.58712
O	9.31143	1.86944	1.05611
O	-0.7537	-11.4866	-2.45913
O	-1.788	-9.32402	-3.23164
O	-10.827	0.25894	1.66423
O	-9.0033	1.54191	0.49229
C	0.30894	9.60216	4.49092
H	-0.5383	10.24731	4.25996
H	1.07085	10.14682	5.05213
H	-0.0093	8.71165	5.03068
C	11.1549	0.07921	1.6433
H	11.7633	-0.67372	1.14466
H	10.452	-0.37608	2.33727
H	11.7843	0.81076	2.15079
C	-0.0809	-10.5541	-4.81832
H	0.83506	-11.1391	-4.75179
H	0.09123	-9.60632	-5.32329
H	-0.8677	-11.1226	-5.31488
C	-10.814	0.29384	-0.95939
H	-11.398	-0.62546	-0.94137
H	-10.114	0.30157	-1.79152
H	-11.468	1.16593	-0.99242

Table S3. Orbital energies in Hartree computed at PBE0/6-311G* level of theory, with C-PCM considering water as the solvent.

	H_4BMet	F_4BMet
**HOMO-4**	-0.25392	-0.27076
**HOMO-3**	-0.25291	-0.27016
**HOMO-2**	-0.24787	-0.26946
**HOMO-1**	-0.20634	-0.22235
**HOMO**	-0.18606	-0.19572
**LUMO**	-0.10000	-0.11217
**LUMO+1**	-0.04812	-0.05957
**LUMO+2**	-0.02048	-0.04004
**LUMO+3**	-0.01961	-0.03864
**LUMO+4**	-0.01849	-0.03790

Determination of Partition Coefficient (P_{OW})

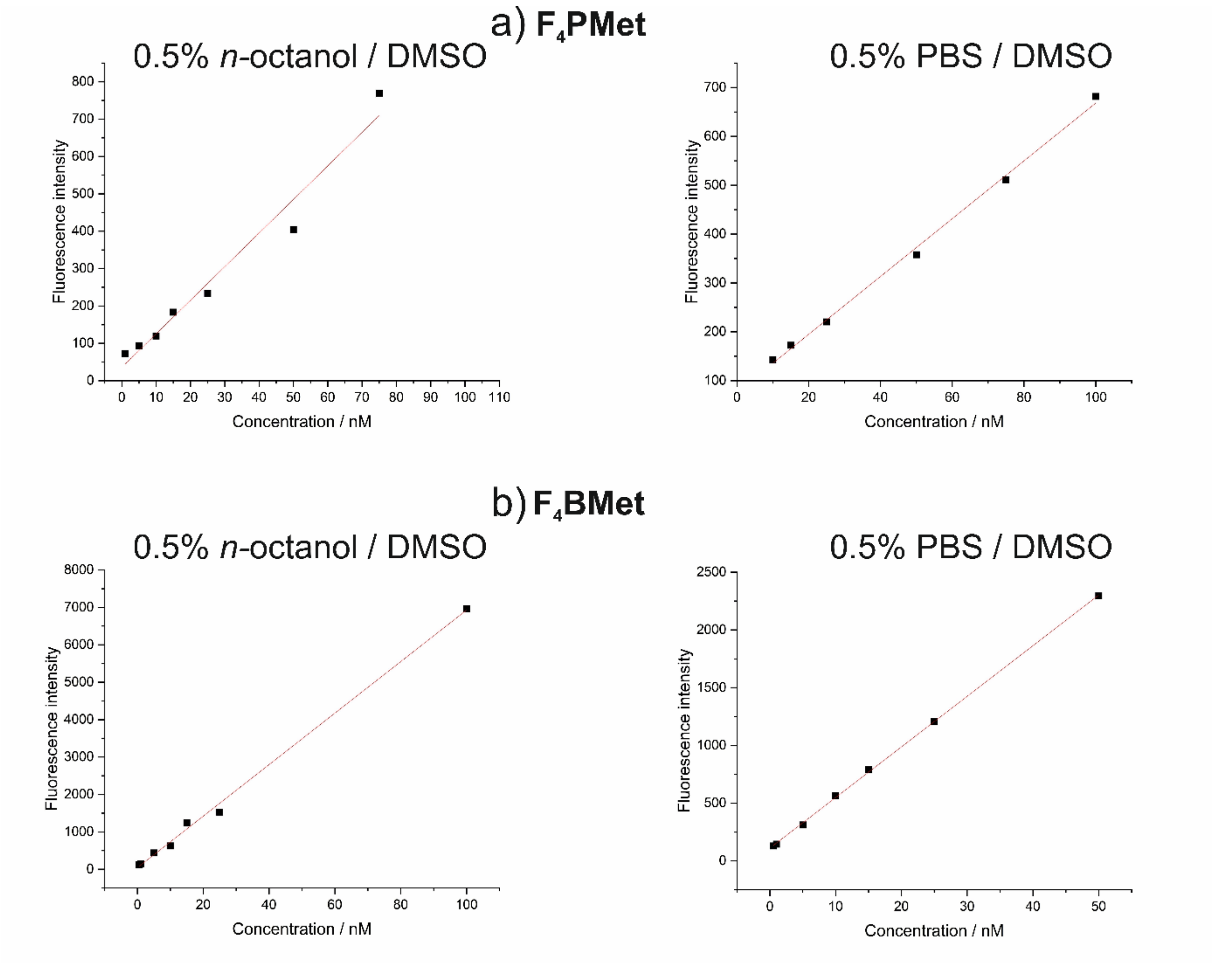

Figure S4. Fitting curves related to the experimental values of LogP for a) porphyrin F_4PMet and b) F_4BMet based on fluorescence intensities of photosensitizers in DMSO containing respectively 0.5% PBS buffer or octanol.

Table S4. Partition Coefficient values were determined for two porphyrins (F_4TPP-MS, F_4PMet) and bacteriochlorin (F_4BMet).

Photosensitizer	LogPow
**F_4TPP-MS**	4
**F_4PMet**	2.26
**F_4BMet**	1.53

Transient absorption spectra of F₄BMet

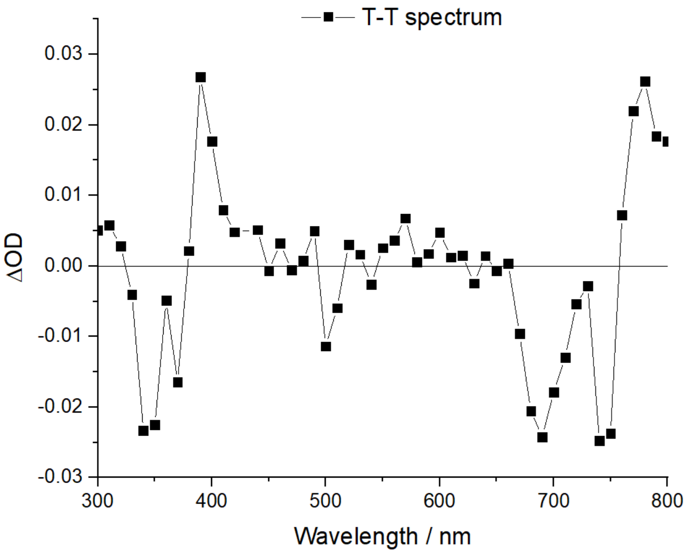

Figure S5. Transient absorption spectra of F_4BMet in water, recorded in the absence of oxygen at 20°C, λ_{exc}=355 nm.

Determination of F₄BMet triplet state lifetimes in the presence and absence of oxygen

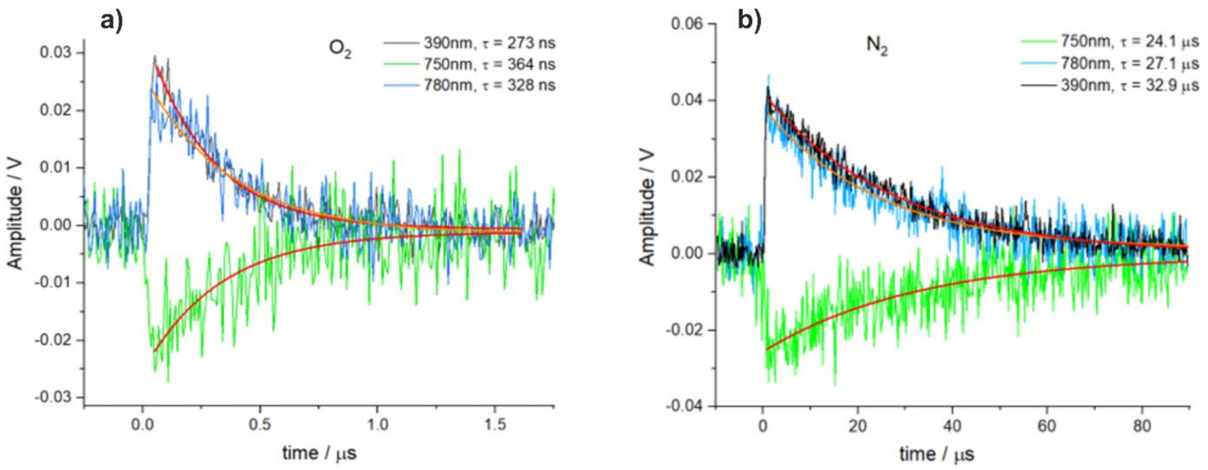

Figure S6. Triplet state decays of the **F_4BMet** in the air-saturated DMF solutions measured at 390, 750, and 780 nm by laser flash photolysis at 20 °C with λ_{ex}=355 nm, measured in the conditions of a) air (O_2), b) air absence (N_2)

Characterization of P123-encapsulated photosensitizers – DLS data

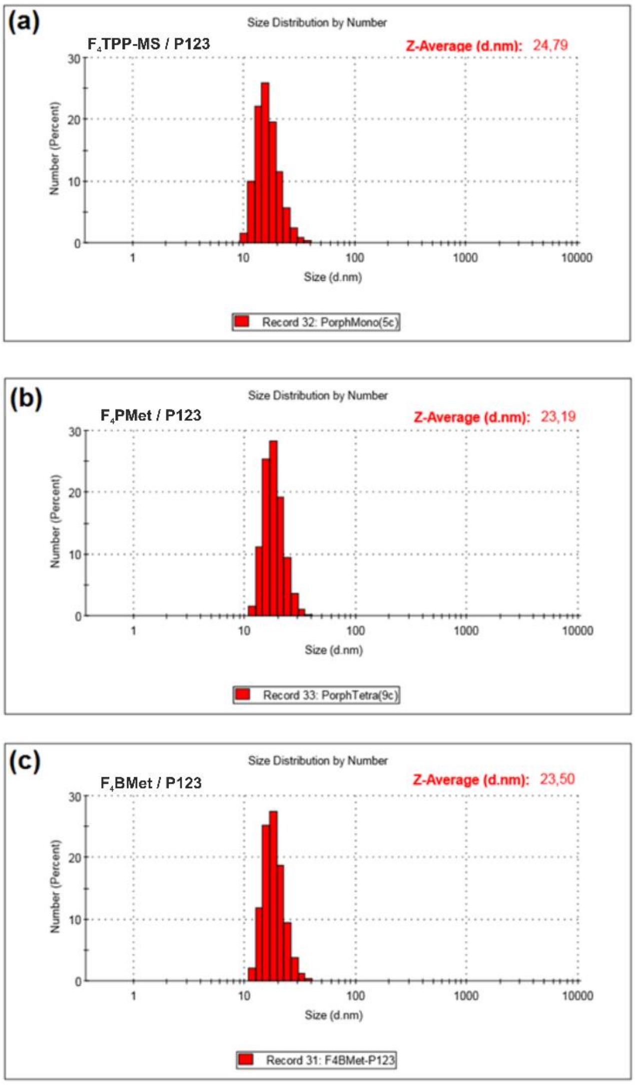

Figure S7. The particle sizes of photosensitizers formulated in Pluronic P123 micelles measured by dynamic light scattering (DLS): (a) **F_4PMet** and (c) **F_4BMet**

Confocal fluorescence imaging of F_4BMet accumulation in CT26 cancer cells

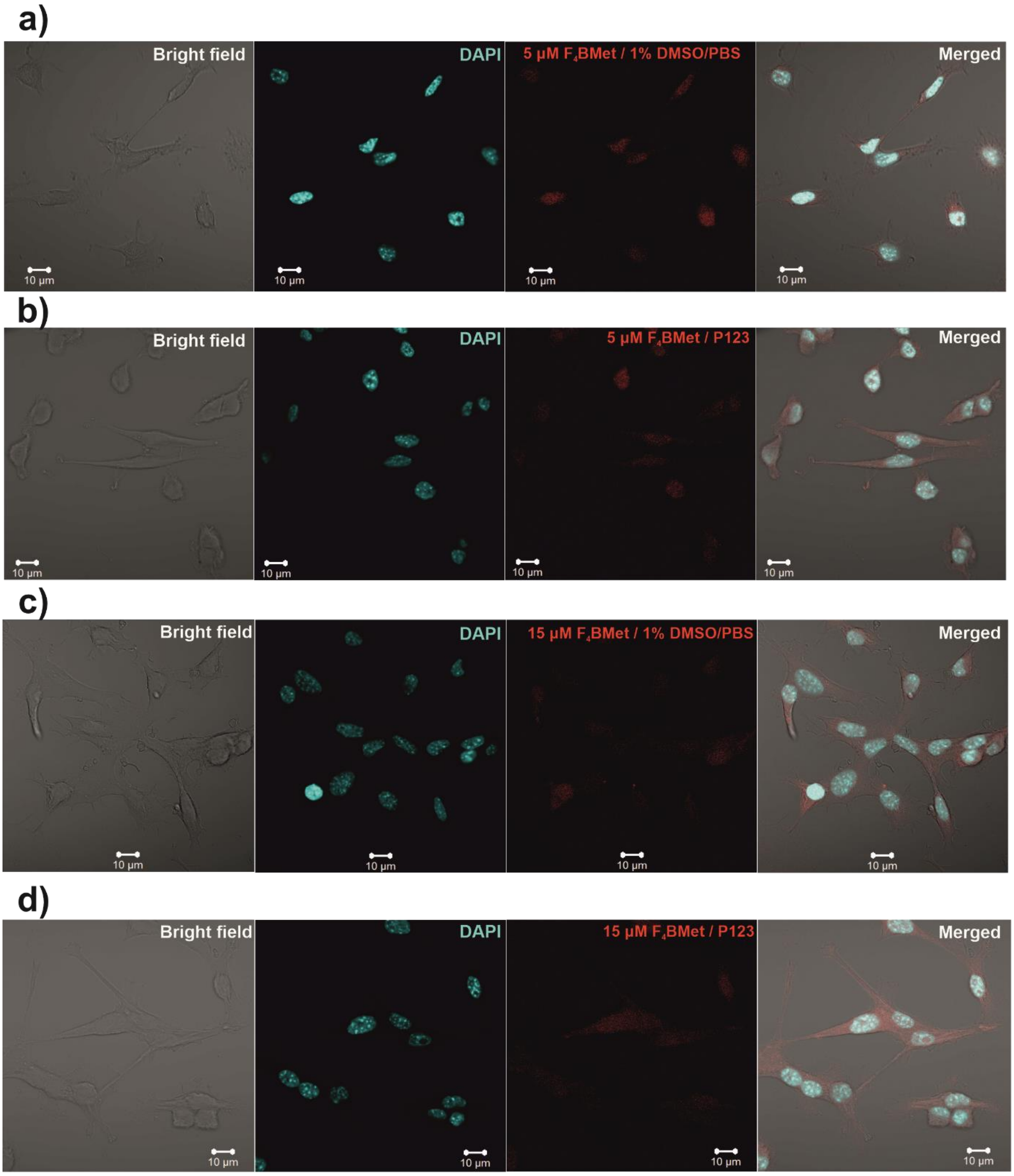

Figure S8. Bright field, fluorescence imaging of nucleus-specific dye DAPI and F_4BMet in two different formulations, and merged images of CT26 cancer cells after incubation with a) 5 µM F_4BMet in 1% DMSO/PBS solution, b) 5 µM F_4BMet in Pluronic P123 micelles, c) 15 µM F_4BMet in 1% DMSO/PBS solution, and d) 15 µM F_4BMet in Pluronic P123 micelles. F_4BMet was excited with 405 nm and emission was observed over 745 nm wavelength.

Changes in cancer cell morphology induced by photodynamic effect with investigated photosensitizers

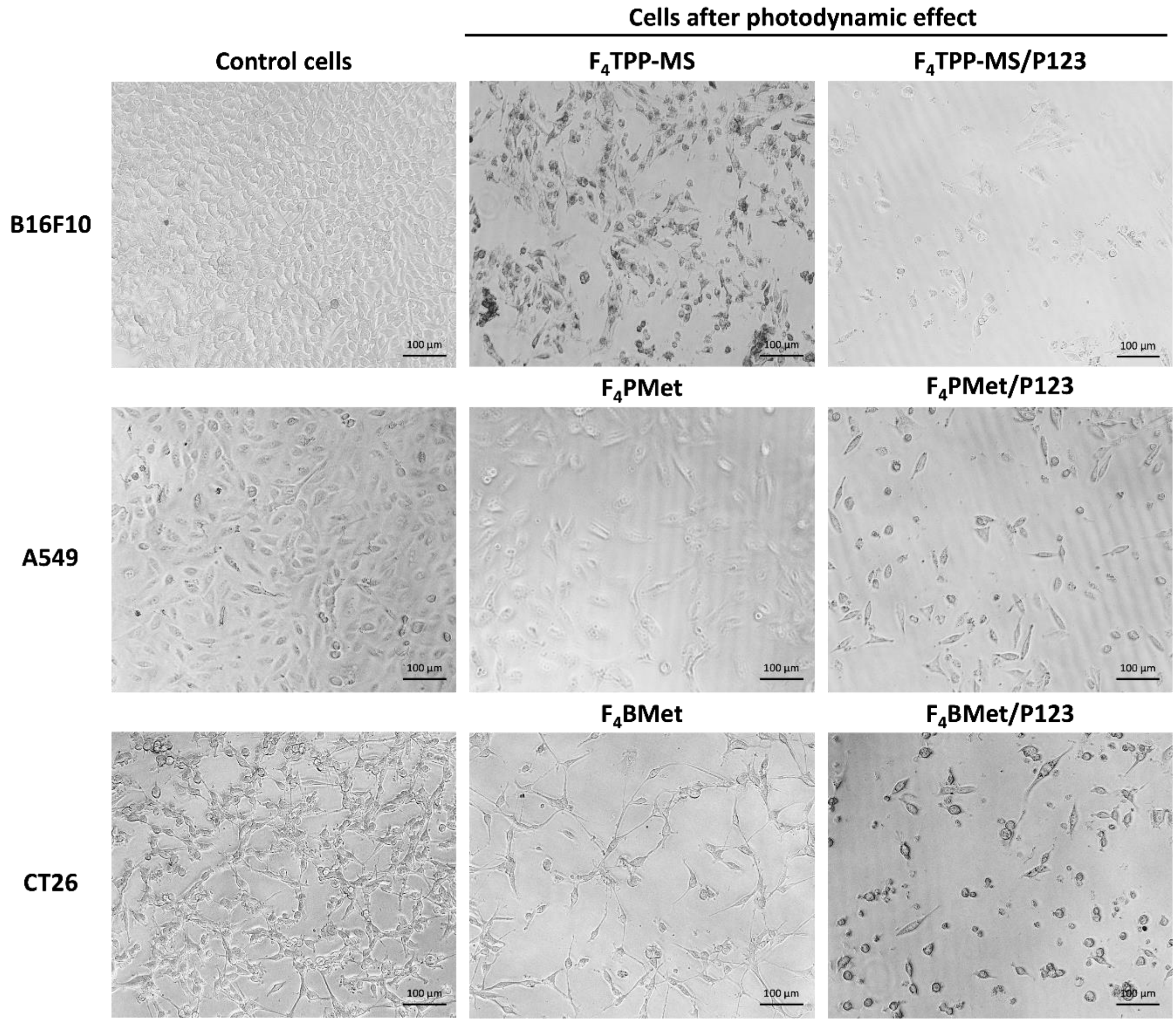

Figure S9. Selected cancer cell morphology before (control cells) and after photodynamic effect with studied photosensitizers without and after P123 encapsulation.

Confocal fluorescence imaging of live/dead CT26 cells before and after F_4BMet / P123 photodynamic therapy with NIR light.

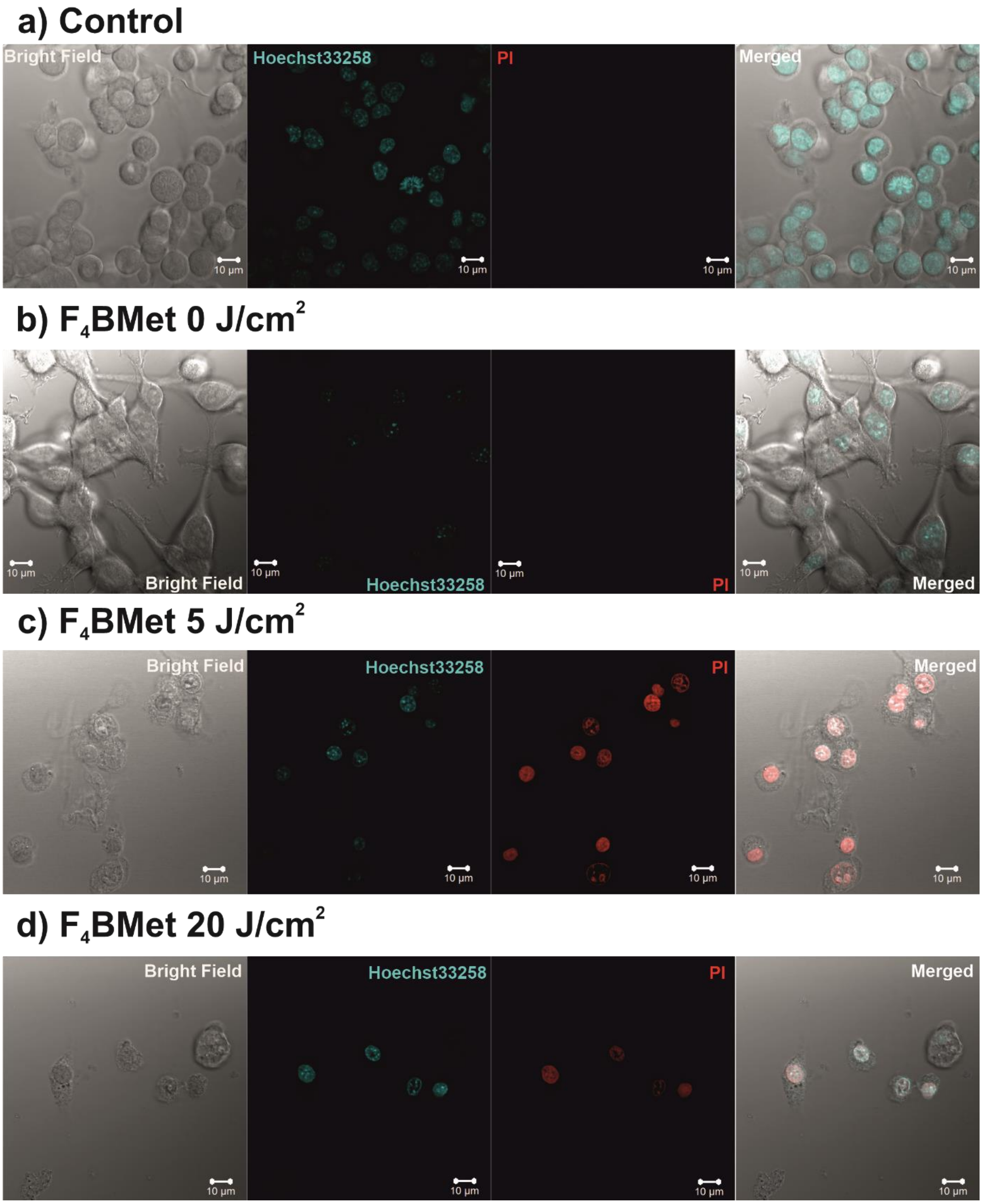

Figure S10. Confocal fluorescence imaging of PDT effect of F_4BMet / P123 against CT26 cancer cell line showed bright field images, fluorescence imaging of nucleus-specific dye - Hoechst33258, dead/destroyed cells dye - propidium iodide, and merged images. Images were taken to show a) control cells, b) cells incubated with F_4BMet / P123 for 24 h without irradiation, c) cells incubated with F_4BMet / P123 for 24 h, irradiated with a light dose of 5 J/cm^2, and d) cells incubated with F_4BMet / P123 for 24 h, irradiated with a light dose of 20 J/cm^2.

Characterization of bacteriochlorin through NMR Spectroscopy

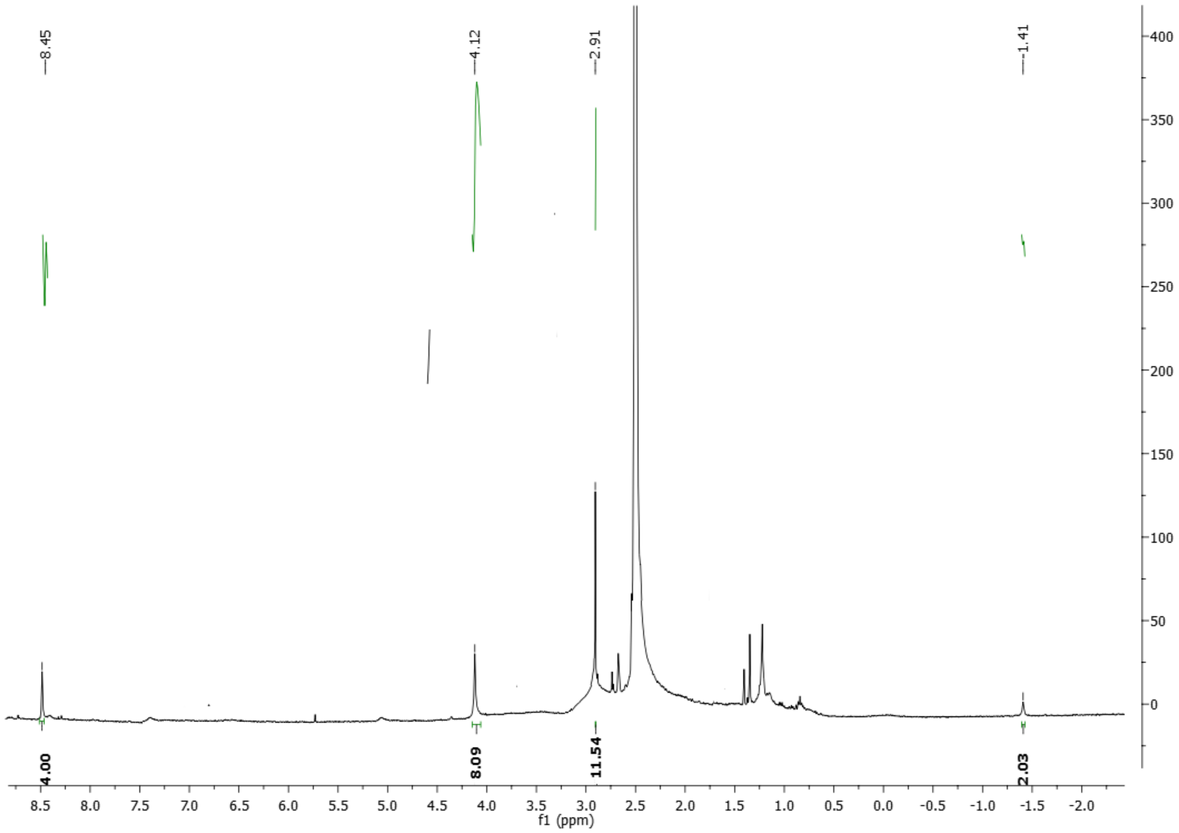

Figure S11. ^{1}H NMR spectra of **F$_4$BMet** in DMSO-d$_6$.

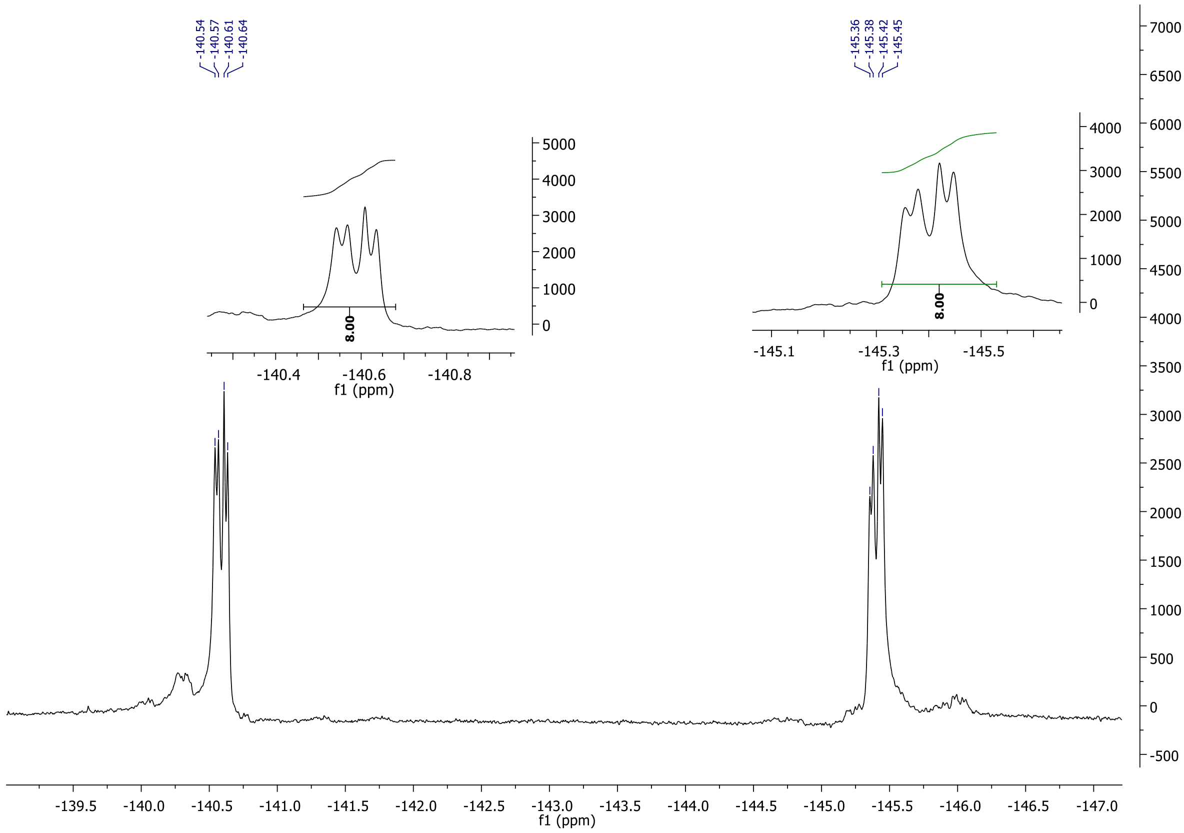

Figure S12. ^{19}F NMR spectra of **F_4BMet** in DMSO-d_6.

Characterization of studied PS by Mass Spectrometry

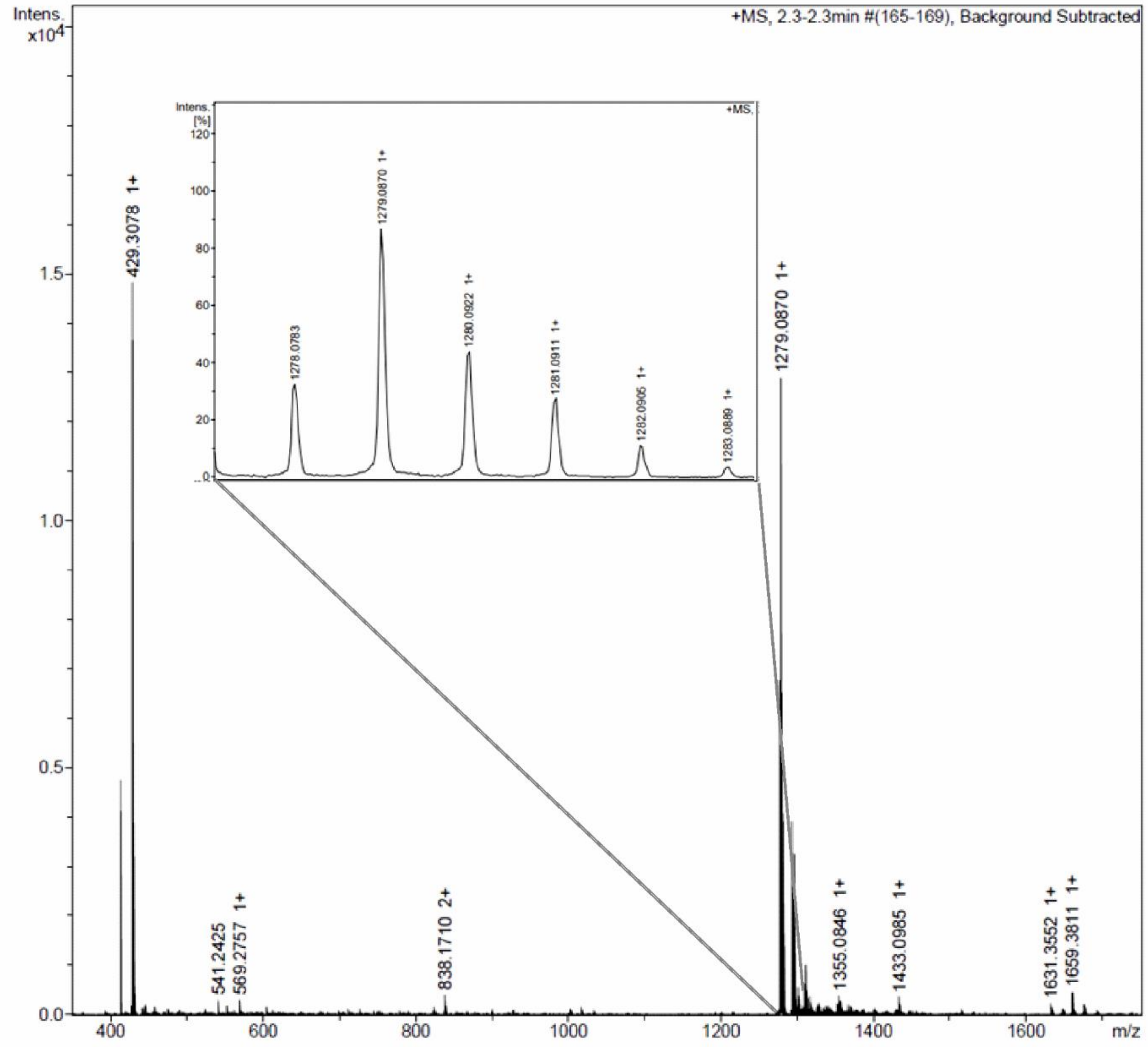

Figure S13. ESI-FIA-TOF Mass Spectra obtained for **F$_4$BMet**.
